# Neuronal responses in the human primary motor cortex coincide with the subjective onset of movement intention in brain–machine interface-mediated actions

**DOI:** 10.1371/journal.pbio.3003118

**Published:** 2025-04-17

**Authors:** Jean-Paul Noel, Marcie Bockbrader, Tommaso Bertoni, Sam Colachis, Marco Solca, Pavo Orepic, Patrick D. Ganzer, Patrick Haggard, Ali Rezai, Olaf Blanke, Andrea Serino

**Affiliations:** 1 Department of Neuroscience, University of Minnesota, Minneapolis, Minnesota, United States of America; 2 Minnesota Robotics Institute, University of Minnesota, Minneapolis, Minnesota, United States of America; 3 Department of Physical Medicine and Rehabilitation, The Ohio State University, Columbus, Ohio, United States of America; 4 MySpace Lab, Department of Clinical Neuroscience, University Hospital Lausanne (CHUV), Lausanne, Switzerland; 5 Department of Clinical Neurosciences, University Hospital, Geneva, Switzerland; 6 Medical Devices and Neuromodulation, Battelle Memorial Institute, Columbus, Ohio, United States of America; 7 Neuro-X Institute, Faculty of Life Sciences, Swiss Federal Institute of Technology (EPFL), Lausanne, Switzerland; 8 Department of Biomedical Engineering, University of Miami, Miami, Florida, United States of America; 9 Institute of Cognitive Neuroscience, University College London, London, United Kingdom; 10 Rockefeller Neuroscience Institute, West Virginia University, Morgantown, West Virginia, United States of America; German Primate Centre Leibniz Institute for Primate Research: Deutsches Primatenzentrum GmbH - Leibniz-Institut fur Primatenforschung, GERMANY

## Abstract

Self-initiated behavior is accompanied by the experience of intending our actions. Here, we leverage the unique opportunity to examine the full intentional chain—from intention to action to environmental effects—in a tetraplegic person outfitted with a primary motor cortex (M1) brain–machine interface (BMI) generating real hand movements via neuromuscular electrical stimulation (NMES). This combined BMI-NMES approach allowed us to selectively manipulate each element of the intentional chain (intention, action, effect) while probing subjective experience and performing extra-cellular recordings in human M1. Behaviorally, we reveal a novel form of intentional binding: motor intentions are reflected in a perceived temporal attraction between the onset of intentions and that of actions. Neurally, we demonstrate that evoked spiking activity in M1 largely coincides in time with the onset of the experience of intention and that M1 spike counts and the onset of subjective intention may co-vary on a trial-by-trial basis. Further, population-level dynamics, as indexed by a decoder instantiating movement, reflect intention-action temporal binding. The results fill a significant knowledge gap by relating human spiking activity in M1 with the onset of subjective intention and complement prior human intracranial work examining pre-motor and parietal areas.

## Abstract 2

Movements can be initiated before or when one becomes aware of the intention to move. This study shows that spiking activity in the human primary motor cortex coincides with the onset of subjective intention, extending prior work on pre-motor and parietal brain areas.

## Introduction

Goal-directed actions and their consequences on the environment are subjectively preceded by motor intention [1]. This same subjective experience can be elicited by direct electrical stimulation of frontal [2] and parietal areas [3], with subjects reporting the “urge to move” upon stimulation. Further, spiking activity from neurons in frontal cortical areas anticipates the subjective experience of intending actions by up to ~1.5 s [4]. These findings suggest that a frontoparietal circuit initiates movements *prior* to the agent being aware of this intention [5–10], fueling a long-term debate regarding the nature of free will. However, the neural mechanisms *co-occurring* with the subjective experience of intending actions remain unknown.

The study of the subjective phenomena accompanying motor intention has largely relied on two paradigms based on time perception: the “Libet task” [5] and “Intentional Binding” [11, 12]. In the former, participants self-initiate movements and report the perceived time of their intention while neurophysiological measures are taken. Libet’s seminal observation [5] was that a slow drift of neural ensemble activity, the readiness potential [13], preceded not only the onset of voluntary actions but also the subject’s perceived timing of intention (but see [14,15] for studies questioning the interpretation of these findings and [16] for a recent review highlighting the difficulty in estimating the timing of subjective intention). In the “Intentional Binding” paradigm [11,12], participants perform a simple action leading to an effect on the environment. Then, participants report on the timing of their action or its consequent environmental effect. The seminal observation [11,12] is that voluntary actions (but not involuntary ones) lead to a compression in the perceived timing between actions and effects.

Studies employing these paradigms have significantly advanced our understanding of motor intention, and have highlighted a common neural circuitry including the anterior cingulate cortex (ACC), supplementary motor areas (pre-SMA and SMA proper), and the posterior parietal cortex leading to self-initiated behavior [5,11,12,17–22]. However, these studies have not examined the full intentional chain; from intention to action to effect. Indeed, the voluntary actions in the “Libet Task” omit environmental effects, while “Intentional Binding” invokes intention, but does not measure it (querying action and effect, but paradoxically not intention). More vexingly, there is a dearth of cellular recordings surrounding intentional processes and examining subjective experience—which is only possible in humans. For instance, “Intentional Binding” has never been examined during invasive single-cell recordings in humans. In turn, the relative timing between the subjective experience of intention, action, and effect, and their associated neural activity remains unclear. Finally, most previous work has focused on preparatory or precursor activity in higher-order areas of the frontal motor hierarchy, yet have failed to consider the contribution of primary motor cortex (M1) in the intentional chain. Primary motor cortex is the last cortical node guiding action—what Sherrington [23] called “the final common path”—and the neural node most targeted in building and deploying invasive brain–machine interfaces (BMIs) to restore motor control. Yet, we do not know if and how M1 reflects subjective intention and its temporal binding to other elements of the full intentional chain.

To bridge these gaps in knowledge, here we leveraged the unique opportunity to examine the full intentional chain in an expert intracortical M1 BMI user who is outfitted with neuromuscular electrical stimulation (NMES) leading to movements of his own, real body [24–26]. This unique setup allowed us to realize a novel intentional chain paradigm in which we systematically enable or disable each element of the intentional chain while recording neural activity in M1 and collecting subjective reports regarding the perceived timing of intentions, actions, and environmental effects.

## Results

### Temporal binding between intention and action

**Fig 1:**
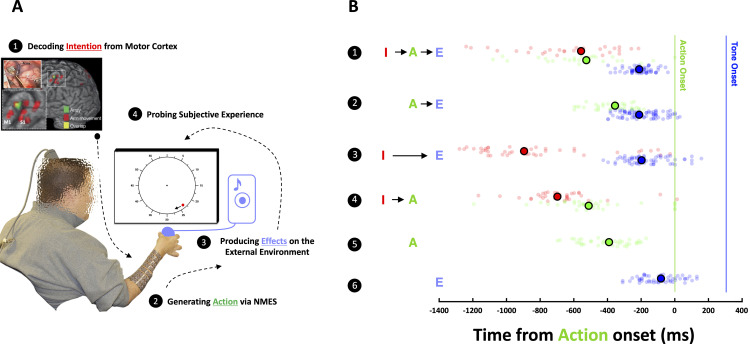
Temporal binding between intention and action. **(A)** Neural recording and experimental setup. Top: T1-weighted magnetic resonance imaging (MRI) showing the overlap between arm/hand movement areas (from functional MRI) in primary motor cortex and the placement of the recording array as indicated by post-operative tomography. Bottom: Rendering of the experimental setup, where the BMI user (pixelated for privacy) viewed a clock with a single hand (red dot) performing a full cycle in 2,560 ms and had to indicate when he first intended to move, when he moved (action), or when tones occurred (effect). **(B)** Behavioral Results. *Row 1*. Full intentional chain where the BMI user indicates time of intention (“I” indicates “intention”, red), action (“A” indicates “action”, green), and an effect (“E” indicates “effect”, blue) in the external environment. Overall, timing is biased (vertical lines are objective timing) but relative timing is accurate and precise. *Row 2*. Estimates of the timing of actions and effects in the absence of intention. *Row 3*. Estimates of the timing of intentions and effects in the absence of actions. *Row 4*. Estimates of the timing of intentions and actions in the absence of an effect. *Row 5*. Estimate of the timing of actions in the absence of intentions and effects. *Row 6*. Estimate of the timing of effects in the absence of intentions and actions. Big, filled circles outlined in black are median estimates, while smaller semi-transparent circles are individual trials. The data underlying this figure can be found at https://osf.io/k8r93/.

The participant was a C5/C6 tetraplegic person outfitted with a Utah microelectrode array (96 electrodes) in the hand region of M1 (Fig 1A). He is an expert BMI user [24–26] able to perform dexterous movements with his real forearm/hand given that appropriate NMES is provided based on neural decoding. To this end, prior to each session, the participant was asked to attempt two movements, right hand opening (HO) and closing (HC). From these, a non-linear support vector machine (SVM, [27]) was trained to detect and discriminate between intended movements (evoking HC when requested: 89.2%; evoking HO when requesting HC: 0%; see Methods as well as S1 Fig and its caption for additional detail). During experimental sessions (total of 12 sessions, ~35 hours total), HCs were associated with an environmental effect (i.e., it was an operant action), and the participant was asked to initiate these actions at his own urge. His intention to move was decoded via the BMI system and NMES was used to realize the action. The participant held a ball in his hand, serving as an apparent actuator causing an auditory tone 300 ms after squeezing it, i.e., an effect in the environment. In different blocks of trials, distinct elements of the intentional chain were bypassed by generating an involuntary hand movement via NMES (i.e., absence of intention), by not activating the NMES when an intention was decoded (i.e., absence of action), or by not generating the sound after HC (i.e., absence of effect). During the task, the participant viewed a rendering of a clock with a single hand that would complete a full cycle in 2,560 ms. The clock had ticks spanning from 0 to 60 (overlapping at “noon”) and marked every 5 units (see **Fig 1A**). After each trial (~2.5–3 s post-movement), the participant was prompted to orally report the timing of a single element of the intentional chain by indicating at which location (i.e., from 0 to 60) was the hand (i.e., red dot in **Fig 1A**) when he either intended the action, actually moved, or the tone was played. The participant was informed prior to each trial which element of the intention chain he would have to report on upon trial completion. The oral report was noted by a hypothesis-naïve experimenter.

Overall, the BMI participant had a bias in the temporal perception of his actions and their consequences, estimating these to occur ~450–500 ms prior to their objective timing (mean ± S.E.M.; action-time: −455 ms ± 21 ms; effect-time; −512 ms ± 17 ms, **Fig 1B**). This bias may stem from long-term sensorimotor recalibration due to the participant’s medical condition and/or training with BMI systems, or could be a consequence of the delay between experience and report (although see [28] for a similar effect in healthy humans, and see the Discussion for important considerations regarding the putative generalization of the effects herein reported given the participant’s condition). Importantly, the relative timing between hand movement and the following auditory cue was accurate (median of all possible permutations of differences between action to effect estimates = 257 ms; objective timing difference = 300 ms) and precise (S.E.M = 17 ms), demonstrating that the participant was able to accurately and precisely observe and report the timing of environmental events. The variance associated with repeated queries of the onset of subjective intention was ~1.5× larger (1 S.E.M = 37.2 ms) than when querying phenomena with an overt physical consequence (action, S.E.M = 21.4 ms; effect, S.E.M = 17.2 ms).

Under the full intentional chain, when intentions led to actions which in turn led to environmental effects, the participant perceived intention to lead actions by 71ms, and the effects to follow actions by 314 ms (error from objective timing = 14 ms. **Fig 1B, Row 1**). When removing intention and instead provoking HCs at arbitrary times, actions were perceived to occur much later in time (with intention: −526 ms ± 44 ms relative to the objective time of action; without intention; −355 ms ± 18 ms, *p* = 9.09 × 10^−5^, **Fig 1B, Row 2).** When removing actions by transiently disabling the NMES command, and instead activating the tone at a fixed delay after the decoder reached threshold for motor execution, intention was perceived to occur much earlier in time (with action: −597 ms ± 79 ms; without action: −796 ms ± 129 ms, *p* = 0.02, **Fig 1B, Row 3**). In neither manipulation—selectively bypassing intention or action—did the estimated timing of the tone (i.e., effect) change (all *p* > 0.1). Lastly, eliminating environmental effects from the full intentional chain did not alter the perceived timing of intentions (*p* = 0.41) or actions (*p* = 0.07) relative to the full intentional chain (**Fig 1B, Row 4**). These results show a novel form of intentional binding; a compression in the subjective timing between intention and action.

Whereas previous temporal binding was observed for the association between two physical events, actions, and effects [11,12], here we describe a new form of binding between a purely internal phenomena (intentions) and a physical action (see S1 Text for a partial replication of the traditional action-effect binding [11,12]). Intention-action binding could have not been revealed in previous work given that only in a participant with a disconnection between brain and end-effectors can intention and motor output be independently controlled and measured.

### Average M1 evoked responses largely co-occur with the experience of intention

**Fig 2:**
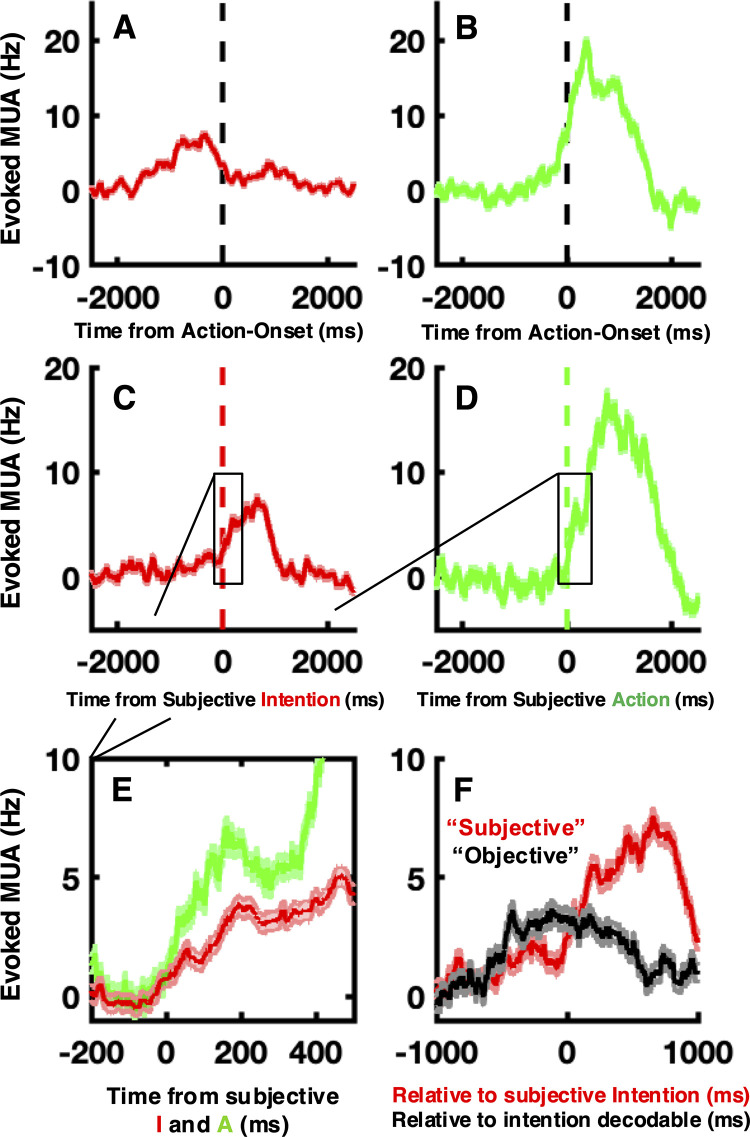
Evoked activity. **(A)** MUA evoked during intention-only trials aligned to the time of decoder crossing threshold for movement. (**B)** MUA evoked during movement-only trials aligned to the time of movement onset. (**C)** MUA evoked during intention-only trials aligned to the report of subjective onset of intention. (**D)** MUA evoked during movement-only trials aligned to the report of subjective motor onset. (**E**) Zoomed-in version (x-axis spanning −200 to 500 ms) of **(C)** and **(D)**, directly contrasting MUA aligned to subjective experience during intention-only and movement-only trials (“I” and “A”, respectively). (**F)** MUA during intention-only trials, aligned to the subjective timing of intention (red, same as in **C**. and **E**.) and to the timing at which the SVM decoder was first able to reliably detect intentionality (“objective” timing of intention and matching on average the relative subjective delay between intention and actions, black). Shaded areas surrounding the average MUA are 1 S.E.M. The data underlying this figure can be found at https://osf.io/k8r93/.

Multi-unit activity (MUA) was aligned to movement onset, or in the case of movement-absent intentional trials, to when movement onset would have occurred given the BMI decoding. Results demonstrated robust evoked MUA in M1 during intention-only trials (peak ~5–8 Hz occurring ~338 ms prior to movement onset, Fig 2A), as well as during NMES-activated movement (peak ~20 Hz occurring ~373 ms after movement onset, Fig 2B). There was no evoked MUA response to tones (S2A Fig). These results demonstrate that intention (but not auditory tones), even in the absence of overt movement, result on average in evoked neural activity in human M1. These intention-only evoked responses precede action-related activity, and are expectedly smaller than action-related activity. Further, a direct comparison of intended versus unintended actions confirms the presence of a gradual rise in firing rate prior to action initiation when actions were intended (S3 Fig, pink and green). When actions were evoked via NMES in the absence of intention, neural activity on average rose shortly before movement onset, an effect that is due to (1) the autocorrelated nature of biological signals and the fact that M1 fires during movement and (2) the need for convolutions (i.e., akin to low-pass filter) to estimate firing rates from discrete spiking activity. Later, at the level of single units (as opposed to multi-unit activity here), we examine whether this gradual rise in firing rate is observed on single trials, and/or whether it may be better characterized as ‘steps’ in activity, as opposed to ramps (see [29–31] for a broader discussion regarding whether decisions reflect continuous ramps or binary thresholds). For now, these results show dissociable signals in M1 related to intention or action (i.e., evoked activity present in intention-only trials, different timing and magnitude for intention- and motor-evoked responses).

Next, we were interested in the temporal relation between this activity and the subjectively perceived timing of events along the intentional chain—an aim solely possible when recording extra-cellular activity and simultaneously requesting a subject to explicitly report on the timing of intentions, actions, and effects. Thus, we aligned MUA to the subjective timings (i.e., reports) of intentions, actions, or environmental effects. Results showed that averaged MUA largely followed the subjective experience, with the onset of evoked responses (defined as +5 standard deviations from baseline) being evident 14 ms after the subjective timing of intention (**Fig 2C**) and 7 ms after the subjective timing of action (**Fig 2D**; see **Fig 2E** for a zoomed-in version directly comparing intention and action). No responses were found to the subjective timing of environmental effects (S2B Fig). We note that these are relative timings between subjective reports (of intention and action in this case) and the onset of evoked and average firing rates and are (1) just averages and not fully descriptive of individual events (see below), (2) depend on an arbitrary definition of the onset of evoked responses, and (3) are corrupted by sources of uncertainty at different scales (e.g., perception of the dot along the clock, drifts in an internal criterion for what constitutes “the experience of intention”, or Poisson variability of spiking activity, to name just a few). Thus, to ascertain a confidence interval over these temporal relationships, we repeated the above analyses while “perturbing” in time individual trial reports by a random amount (uniform distribution of increasing widths, from 10 ms to 1,000 ms in steps of 10 ms) and ask: when are evoked responses significantly degraded? Our estimate is that evoked responses (both to intention and action) remained robust for temporal perturbations of up to ~120 ms (S4 Fig).

Quite uniquely, within this BMI context, we could also determine on a trial-by-trial basis an “objective” timing of intention. We operationalized the latter as the first time point within a trial wherein the BMI decoder surpassed 5 standard deviations above its noise level. Note that this criterion is more liberal than that for motor initiation, and on average precedes motor execution by ~70 ms. This matches the average subjective delay between intention and action during the full intentional chain (**Fig 1B, Row 1**), but on a trial-by-trial basis differs from the reported—subjective—timing of intentions. Aligning MUA during no-movement trials to both “objective” and “subjective” timing of intention showed that average evoked activity in these trials largely coincided with the subjective report of intending an action (**Fig 2F**, red), but not with the “objective” timing of this intention (**Fig 2F**, black), as indexed by the BMI-decoder. This argues that while decoders (either artificial or downstream neural areas) may have access to an anticipatory or preparatory “intention-signal” (e.g., in SMA or ACC; REF 4), the subjective experience of intention largely co-occurs (± ~120 ms) on average with the onset of robust evoked spiking activity in human M1.

### Temporal relationship between population dynamics in M1 and subjective onset of intention

To examine the average temporal relationship between the subjective experience of intention and population dynamics, we projected spiking activity for all 96 channels onto their first two principal components. This accounted for 81.9% of the total variance. The contrast between conditions differing solely by the presence (versus absence) of intention showed that trajectories in a low dimensional latent space bifurcated (S5A Fig). The distance (Euclidian in 2D) between these conditions gradually grew prior to movement, peaked 300 ms post movement onset, and then precipitously dropped (S5A Fig; see S5B Fig and S5C Fig for the same analyses for trials missing either actions or environmental effects). We further investigated whether population dynamics in M1 reflect subjective experience by testing whether they discriminate between trials matched for the presence of intention, action, and environmental effects, but differing in their subjective temporal onset. The contrast between trials where motor intention was perceived relatively early versus late differed between −1,360 ms and −360 ms post-movement onset (S6A Fig, see S6B Fig and S6C Fig for the same analysis examining timing of actions and effects).

Similarly, we examine if and how conditions differing by the presence or absence of a single element of the intentional chain (intention, action, effect) differed at the level of power spectra in different frequency bands of the local field potential. The presence of intention appeared to be reflected in sustained power in the delta band (0.5–4 Hz) and perhaps more intermittently (or varied across trials) in theta (4–8 Hz) and alpha (8–13 Hz) bands (S7A Fig). Indeed, while power in these lower frequency bands were present even before movement in conditions with intention, they were only induced after movement when NMES-activated actions were provoked in the absence of intention (S7A Fig, rows 1, 3, and 4 versus 3). Movement was most readily reflected by a transient increase in power in the alpha and lower beta bands (S7A Fig, rows 1, 2, and 4 versus 3). To further quantity these effects (and draw parallel to the decoding used to evoke movements, see below), we trained an SVM classifier ([27], 10-fold cross-validation) to establish the degree of separability between baseline (−2,200 ms to −2,000 ms) and induced power (from −2,000 ms to 2,000 ms post-action, in steps of 100 ms). We then compare this classification accuracy in the full intentional chain relative to conditions lacking a single element (S7B Fig, red = intention; green = action; blue = effect). Results show that conditions differing by the presence versus absence of intention are most separable in lower frequency ranges (delta, theta, alpha) with significant differences across conditions (*p* < 0.01) emerging as early as 1.09s (delta), 0.98s (theta), and 0.87s (alpha) prior to movement. The peak difference between conditions with and without intention occurs 290 ms and 210 ms prior to movement in the delta and theta bands, and co-occurs with movement onset in the alpha band (S7B Fig). Movement decoding (relative to baseline) is most prominent in alpha and beta bands and peaks after movement onset (~330 ms post-movement for both the alpha and beta bands).

Overall, these results demonstrate an interesting dichotomy between linearly and independently averaged spiking activity (MUAs) and population dynamics (PCA or LFP, which by definition incorporate covariance information). While the former largely co-occurs with the subjective onset of intention (**Fig 2**), the latter (1) index intention up to ~1 s prior to movement, and (2) show strongest correlates of intention within a ~200–300 ms window surrounding movement onset. Importantly, even in low frequencies of the LFP domain (i.e., the slowest evolving dynamics), intention is not evident in M1 as early as it may be detected in ACC, SMA, and pre-SMA ([4], ~1.5 ms prior to movement).

### M1 reflects the timing of intentions on a trial-by-trial basis

**Fig 3:**
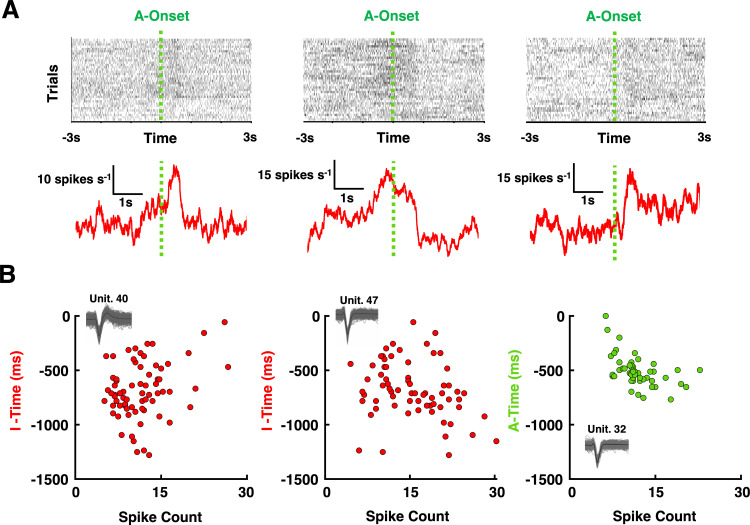
Single cell correlations. **(A)** Example units—raster plots and PSTH. Top: The raster plots show every trial as a row and each black dot is a spike. Bottom: Evoked response surrounding the time of action (“A-onset”) onset for the three representative neurons. (**B)** Correlation between spike counts and subjective estimates of intention or action onset. Depiction of three representative correlations between the subjective timing of intentions (red, examples 1 and 2, “I-time”) and actions (green, example 3, “A-time”) relative to movement onset, and the spike-count of the neuron in the second prior to movement. No correlation is shown for the subjective timing of the tone, as none existed (8/66 for intention, 1/66 for action). The data underlying this figure can be found at https://osf.io/k8r93/.

Next, we questioned whether well-defined single neurons may track the subjective timing of intentions and actions on a trial-by-trial basis. In a first approximation, we restrict this analysis to the first day of recording to assure that single units would not be included in duplicates (i.e., across sessions). Thus, here we examined 66 well-isolated units (examples shown in **Fig 3A**).

We performed a spike count during the 11,000 ms-period preceding actions. Eight neurons (12% out of 66) showed a significant correlation between their spike counts within this period and the reported perceived timing of intentions (all *p* < 0.05 see **Fig 3B, first** and **second column**). All eight neurons are shown in S8 Fig. We note that Fried and colleagues [4] report ~17% of neurons *responding* to intention (as opposed to *co-varying in time* with intention here) in frontal areas, and ~8% in temporal regions [4]. Surprisingly, only a single neuron in the present study showed a correlation between its spike counts during this period and the subjective timing of actions (**Fig 3B, third column,** *r* = −0.62, *p* = 0.01). We did not observe any neuron whose spike count correlated with the perceived timing of environmental effects (see S9 Fig for similar results across different time periods and including neurons recorded in all sessions, not solely the first one). Together, these results suggest that a fraction of single units in M1 may predict the onset of the experience of intention.

We also examined if, when, and how rapidly single unit firing rates on individual trials indexed an upcoming “urge to move” (recall, the report of this timing occurs seconds later, at the end of trials). To do so, we fit a logistic function (Eq 1) of the form,


$$f\left(t\right)=\frac{f_{I}}{1+\exp\left[-\frac{\left(t-t_{o}\right)}{\alpha }\right]}+f_{b}$$ (1)

to individual trial firing rates as they approach the reported intention-time (see [4] for a similar approach). and are respectively asymptotic firing rates during baseline and at the onset of the subjective experience of intention. and are parameters dictating (1) the location (i.e., time) of the central point of the sigmoidal curve describing firing rates, and (2) the steepness of this function (inversely proportional to ). In other words, dictates when firing rates change with respect to the onset of subjective intention, and dictates how quickly this firing rate changes (e.g., from “ramp-like” for a large to “step-like” for small , see [29–31]). All four parameters were fit to individual units and trials, and we retained 177 trials with > 0.5 (see S10A Fig for example individual trials and average responses from 4 units). On average, these units increased in firing rate 108ms prior to the experience of intention (; see S10B Fig for the full distribution), or approximately 700–800ms after a similar effect occurs in SMA, pre-SMA, and ACC [4]. Similarly, the change in firing rates in M1 are more “step-like” (i.e., smaller ) than what is observed in frontal areas (mean = 23 in M1, and mean = 54 in [4], see S10B Fig for the full distributions).

### M1 population dynamics reflect intention-action temporal binding

**Fig 4:**
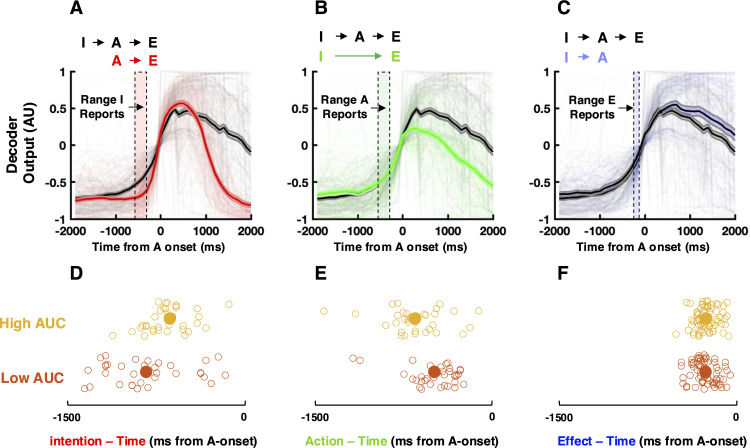
Population dynamics reflect intention-action binding. **(A)** Effect of intention. Contrast of the decoder time-course during the full intentional chain (“IAE”, black) and a chain without intention (“AE”, red) suggests that intention causes an early and gradual rise toward the movement threshold (y = 0). Shaded vertical area enclosed by dashed lines is the median ± 95%CI of the subjective timing of intentions (“I-time”). AU refers to “arbitrary units”, given that the decoder output was normalized between −1 and 1, with the movement threshold being zero-crossing. (**B)** Effect of action. Contrast of the decoder time-course during the full intentional chain (black) and a chain without action (green) suggests that the decoder output decreases shortly after crossing movement threshold when no motor output occurs. Shaded vertical area enclosed by dashed lines is the median ± 95%CI of action reports. (**C)** Effect of tone. Contrast of the decoder time-course during the full intentional chain (black) and a chain without a consequence to movement (blue) suggests that the absence of tone results in a sustained heightened decoder output. Shaded vertical area enclosed by dashed lines is the median ± 95%CI of environmental effects reports. Shaded area around the decoder output is ± 1 S.E.M. All trials are shown in transparent in the background. (**D)** Decoder performance and perceived time of intention. Trials where the area under the decoder curve (AUC) was high (yellow, meaning the decoder was performed well) resulted in the perceived timing of intention to occur later (closer to action) in contrast to trials with a low AUC (orange). (**E)** Decoder performance and perceived time of actions. Trials where the AUC was high (yellow) resulted in the perceived timing of actions to occur earlier (closer to intention) in contrast to trials with a low AUC (orange). (**F)** Decoder performance and perceived time of effect. Trials where the AUC was high (yellow) versus low (orange) resulted in similar perceived timing of the tone. The data underlying this figure can be found at https://osf.io/k8r93/.

Lastly, we look at the impact of each of the elements of the intentional chain on the behavior of real-time BMI neural decoding. First, we contrasted the decoder time-course during the full intentional chain to conditions missing a single element of the sequence. **Fig 4A** shows that when actions were intended, there was a gradual rise toward the movement initiation threshold. Inducing a non-intended movement through NMES also affected the BMI classifier, but through a delayed and more abrupt rise toward threshold. These two conditions (i.e., intended versus not) strongly bifurcated 1,100 ms prior to movement (*p* < 0.01; time of actions at 0 ms), a timing that comfortably precedes the median timing of perceiving intentions during the full intentional chain (−597 ms ± 79 ms relative to A-time). When intention did not lead to action, the decoder rose to threshold normally, but plummeted earlier (*p* < 0.01, 100 ms post-movement onset, **Fig 4B**). Lastly, during the condition lacking environmental effects, the decoder was sustained for a longer period (**Fig 4C**, *p* < 0.01, 500 post-movement onset). These results suggest that the decoder, and thus population dynamics, (1) can index intention to move and (2) that it is sensitive to all aspects of the intentional chain.

Next, we questioned whether performance of the decoder may reflect intention-action binding (see **Fig 1**). As a measure of decoder performance, we used the area under the curve (AUC) of the decoder, as this measure collapses performance over time, and thus reduces the total number of potential comparisons (e.g., decoder performance at each time point versus behavior). This curve is also normalized between −1 and 1 (**Fig 4A**–**4C**), hence controlling for potential spurious effects that could be caused by variations in raw decoder performance across sessions and days.

We coalesced all trials of a particular judgment type (i.e., reports on intention, action, or environmental effect). Then, we computed the AUC of the decoder for each trial and kept the top (high; yellow) and bottom (low; orange) third of trials defined by AUC. Examining the participant’s reports—sorted by AUC of the decoder and independently from behavior—showed that in trials with high AUC (yellow), intention was perceived as occurring later than on trials with low AUC (orange, **Fig 2D**, *p* = 3.77 × 10^−4^). Applying the same approach to judgments regarding the timing of actions, we found the opposite pattern: actions were perceived as occurring earlier (**Fig 2E**, *p* = 0.01) in high (yellow) versus low (orange) AUC trials. In other words, on trials where the BMI decoder more reliably evoked the intended movement (i.e., HC, higher AUC), there was mutual attractive pull between intentions and actions. The perceived timing of effects was not modulated by AUC of the decoder (**Fig 2F**, *p* = 0.22, see S1 Text and S11 Fig for a further control).

Together, these results suggest that M1 population dynamics, as indexed by the multivariate decoder the participant uses to move, reflects intention-action temporal binding. In turn, the behavioral results (**Fig 1B**) argued that actions within a full intentional chain result in a perceived temporal binding between intentions and actions.

## Discussion

We leveraged the unique opportunity to selectively bypass intentions, actions, or the corollary consequences of these actions in a tetraplegic person fitted with an intracortical M1 implant and NMES leading to movements of his own body. This allowed us to index for the first time the perceived temporal binding between all elements of the intentional chain while simultaneously performing extra-cellular recordings in a human. Further, it allowed us to fill a critical gap in knowledge: while prior seminal work indexed the temporal relation between the onset of intention and spiking activity in pre-motor [4] or parietal regions, the temporal relation between M1 spiking activity and the subjective onset of intention remained unknown.

Behavioral results demonstrated a novel form of intentional binding, not between actions and effects [11,12], but between intentions and actions. The time distortions caused by intention-action binding are stronger than those engendered by action-effect binding, which likely reflects long-term and permanent associations between intending and performing an action, in contrast to the more sporadic association between actions and a specific auditory consequence (see S1 Text). Importantly, it must also be noted that this intention-action binding may be amplified in the current participant by virtue of the BMI-based control of actions. Hence, it will be important to confirm these behavioral results in neurotypical subjects employing non-invasive BMIs. Neurally, results demonstrate that unit and population-level dynamics in M1 index the presence of intention. Further, spiking activity largely co-occur (on average, with an acknowledged degree of uncertainty) with the onset of the subjective experience of intention, and spike counts of subset of the population may co-vary with the onset of the subjective experience of intention on a trial-by-trial basis. Population dynamics (which by definition index a large ensemble of units and may also be detected by non-invasive methods) show a looser temporal association between neural activity and the subjective onset of intention.

That M1 reflects intention is implied by earlier work [24–26] demonstrating the ability to detect motor intention in this area. It is also perhaps not surprising, given that M1 is the final central nervous system node in the motor pathway [23]. In contrast, the determination of the temporal relationship between the onset of intention and evoked activity in M1 is a novel and important one, particularly in the context of prior work. Namely, Fried and colleagues [4] showed that neurons in the pre-SMA, SMA, and ACC all anticipate the subjective timing of intention by about 700–1,500 ms. Here, we close a critical gap in knowledge by recording from M1, an area downstream from those previously probed in humans. We show that evoked multi-unit spiking activity in M1 on average largely co-occurs with (± ~120 ms) and does not precede (as in pre-SMA, SMA, and ACC) the experience of intention. The fact that there are direct synaptic connections between pre-SMA/SMA and M1, and yet their relation to the onset of subjective intention differs by ~700–1,500 ms implies that the subjective experience of intention may be mechanistically engendered by a relatively slow accumulation process. Indeed, in this line, it has recently been shown that a fraction of neurons across the entire brain accumulate evidence toward a decision/action on a relatively slow temporal scale and that this accumulation occurs in a “movement-null” subspace [32]. Then, almost instantaneously this neural activity is projected onto a “movement-potent” subspace, enacting movement [32] (also see [33,34]). Putatively, may explain why we may accumulate evidence without causing movement, why there is a fairly large delay between intention-related signals in pre-SMA/SMA/ACC and M1, and why the temporal relation between the onset of subjective experience and neural activity in M1 may differ between evoked MUAs (i.e., perhaps a movement-potent subspace) and higher-dimensional population dynamics (i.e., perhaps a movement-null subspace). The speculation is that the experience of intention may co-occur with a re-formatting of neural activity in M1; from a movement-null to a movement-potent subspace. This speculation is in line with our findings suggesting that while (multivariate) signals related to intention may be present (i.e., are decodable, **Fig 4**) in M1 and elsewhere [9,24–26,35–41] well before the onset of the subjective experience of intention, this latter one largely coincides with evoked MUA activity in M1. This observation, along with our emphasis on relating neural activity with the subjective onset of intentionality, breaks with the dominant effort to ascribe intention at ever-earlier stages of the cortical action hierarchy [42–45].

We do not claim that intention does not originate at earlier nodes—it undoubtedly does—nor we claim that M1 is the necessary and sufficient area for intention. We simply note that the subjective experience of intending an action seemingly largely co-occurs with evoked activity in M1, while it tends to follow neural activity in other pre-motor and parietal areas. In the present study, we could not record from other important sites for intention, such as anterior cingulate, supplementary motor areas, pre-motor, or parietal cortex, as electrodes placement is dictated by clinical and not experimental purposes. Multisite implants or integration of results from multiple patients with electrodes in other sites may contribute to gather a more comprehensive view. It must also be explicitly stated that our findings are correlational in nature. In future experiments, it will be interesting to attempt direct, causal perturbations approaches (such as stimulation, as in [2,3]). These perturbation approaches will likely be most informative with concurrent recordings, such that we may estimate if perturbations are “on-manifold”, or orthogonal to subspaces that eventually lead to movement. Likewise, we must also explicitly acknowledge the temporal uncertainty that exists with reporting the onset of any subjective experience, and in particular the experience of “an urge to move” [16]. Lastly, we must emphasize that the observations we report herein are from a single tetraplegic patient and a skilled BMI user who may have experienced a large degree of neuroplasticity, learning, and sensorimotor adaptations. That these important biological mechanisms are likely at play in the current patient is highlighted by the fact that he shows a large bias in the temporal perception of his actions. However, we must also note that in the current setting, a large delay between “objective” and “subjective” estimates of elements within the intentional chain may have aided in establishing neural correlates of the subjective experience of intention. Indeed, intention is typically too tightly linked to the true onsets of motor actions, thus challenging the parsing of motor intention from motor acts in normal conditions. For now, we must emphasize that it will be critical for future work—including work employing more causal manipulations—to ratify (or not) the current observations, and highlight that there is prior work (albeit of more indirect nature, such as employing scalp electroencephalography, transcranial magnetic stimulation, or work in patients with widespread lesions) both supporting [46] and questioning [47,48] the role of M1 in motor intentions. We must also highlight a recent report [49] demonstrating the generalizability of findings from the current patient to a wider healthy population undergoing scalp EEG.

In conclusion, we show a novel form of temporal binding between the perceived time of intentions and actions and demonstrate that M1 evoked activity (i.e., a linear operation) largely coincides in time (± ~120 ms) with the onset of the subjective experience of intention, may predict trial-to-trial fluctuations in this onset, and reflects perceived intention-action binding. Instead, population dynamics in M1 show a looser association with the subjective onset of intention, preceding the subjective experience by 200–300 ms, but not 700–1,500 ms, as in ACC, pre-SMA, and SMA [4]. Together, these results close a critical gap in our knowledge describing the temporal relationship between neural activity in M1 and the onset of intention.

## Materials and methods

### Ethics statement

Approval for this study was obtained from the US Food and Drug Administration (Investigational Device Exemption) and The Ohio State University Medical Center Institutional Review Board (2013H0164). The study met institutional requirements for the conduct of human subjects and was registered on the http://www.ClinicalTrials.gov website (identifier NCT01997125). The participant referenced in this work completed an oral and written informed consent process before taking part in the study. He also provided written permission for photographs and video. This study was conducted according to the principles expressed in the Declaration of Helsinki.

### Participant

The participant was a 27-year-old male with stable, non-spastic C5/C6 quadriplegia from a cervical spinal cord injury (SCI) sustained 8 years prior to the current experiment. The participant’s International Standards for Neurological Classification of SCI neurologic level is C5 (motor complete) with a zone of partial preservation to C6 [50]. He is an expert BMI user, with over 5 years of usage [24]. The subject has full range of motion in bilateral shoulders, full bilateral elbow flexion, a twitch of wrist extension, and no motor function below the level of C6. His sensory level is C5 on the right (due to altered but present light touch on his thumb) and C6 on the left. He has intact proprioception in the right upper limb and the shoulder for internal rotation through external rotation, at the forearm for pronation through supination, and at the wrist for flexion through extension. Proprioception for right digit flexion through extension at the metacarpal-phalangeal joints was impaired for all digits.

### Surgical procedures and data acquisition

The patient underwent a left frontoparietal craniotomy for implantation of a Utah microelectrode array (Blackrock Microsystems Inc, Salt Lake City, Utah) in the (dominant) hand region of primary motor cortex. This region was identified via pre-operative functional Magnetic Resonance Imaging where the patient was asked to imagine performing hand movements (**Fig 1**). The area was targeted via an intraoperative navigation system (see [24] for details). The array contained 96 channels (4.4 mm × 4.2 mm × 1.5 mm in depth) and was implanted into the cortex using a pneumatic inserter. Reference wires were placed subdurally. Neural data were sampled at 30 kHz and hardware band-pass filtered between 0.3 Hz and 7.5kHz (3rd order Butterworth). Once digitized, the data were transmitted to a central computer running MATLAB (Mathworks, Natick, MA), where signal artifacts due to NMES were removed by blanking over 3.5 ms around the artifact (defined as signal amplitude >500 μV simultaneously in at least 4 of 12 randomly selected channels).

### Movement decoder

A non-linear SVM was used to translate neural activity to intended hand movements. The inputs to the SVM were mean wavelet power (MWP) over 100 ms bins. That is, neural activity was decomposed into 11 wavelet scales (Daubechies wavelet, MATLAB), and the coefficient of wavelets 3–6, corresponding to the multi-unit frequency band spanning from 235 to 3.75 kHz, were then normalized and averaged within 100 ms bins resulting in 96 values, one for each channel. The processed features (i.e., mean wavelet values) were then used as input to a support vector machine algorithm that used sparsity optimization to improve decoder accuracy by zeroing out the least valuable MWP features. Training data for the decoder were obtained by prompting the participant at the beginning of each recording session to imagine performing one of two specific hand movement; HO or HC using an animated virtual hand displayed on a computer monitor. The subject performed seven blocks of classifier training (five repetitions per hand movement type) prior to starting the main experiments (S1 Fig). Training took approximately 10 min per session. Two decoders were built (HO versus rest and HC versus rest), and output classes were computed for each movement that had scores ranging from −1 to 1 (arbitrary units). A score of −1 indicated that the classifier estimated the subject to be at rest, a score of 1 indicated that the classifier estimated the participant to perform an action (see [24–26] for additional detail). During experimental sessions, only HC were used (i.e., operant movement resulting in a consequence in the environment), but two movements were trained during decoding to assure detection and discrimination of intention. The appropriate NMES (see below) became active when the output score for a given movement crossed zero (threshold crossing).

### Neuromuscular electrical stimulation

A NMES system was used to evoke hand movement by stimulating forearm muscles. The NMES system consisted of a multi-channel stimulator and a flexible, 130-electrode, circumferential forearm sleeve. The coated copper electrodes were 12 mm in diameter, spaced at regular intervals in an array (22 mm longitudinally × 15 mm transversely), and delivered current in monophasic, rectangular pulses at 50 Hz (pulse width 500 μs, amplitude 0–20 mA). Desired hand movements were calibrated at the beginning of each session by determining/confirming the intensity and pattern of electrodes required to stimulate intended movements. This procedure took approximately 5–10 min per session.

### Experimental design

From a baseline rest position the subject was asked to perform two movements, right HO and HC. Hand closing resulted in an auditory tone being played 300 ms after movement onset (or the decoder surpassing movement threshold, see above). The analyses in the current report focus on HC trials, as these were associated with consequences in the external environment (i.e., the tone), and thus had all components of a full intentional chain. While performing these movements, the subject observed the rendering of a clock with a single hand that would complete a full cycle in 2,560 ms [5]. The initial position of the clock hand was random on each trial. Over the course of 12 separate sessions (~2–4 hours/session, including setup) the participant completed five different experiments, containing variations where he reported either the time of intention onset, the time of action onset, or the time of sound onset.

In the first experiment, on different blocks, we randomly activated the NMES resulting in HC, or we presented the tone at a random time without NMES (50 repetitions per condition). This experiment was a baseline experiment to assess the subject’s temporal perception of movement and auditory stimuli in the absence of any causal structure (i.e., no intention to move, nor a movement causing the effect in the external environment). The second experiment was the “full intentional chain” where intention resulted in movement, and a particular movement (HC, but not HO) resulted in a tone being played. On different trials, the subject reported the timing either of his intention, movement onset, or tone onset (50 repetitions per condition). The third experiment broke the expectation that HC results in an external consequence, and on different trials the subject was asked to report the timing of his intention to move or actual movement (50 repetitions per condition). In the fourth experiment, the subject was instructed not to imagine moving, and instead at a random time, we activated the NMES resulting in HC. Thus, in this experiment there was movement and an effect to this movement, but no intention. On different trials, the participant indicated either movement or tone onset (50 repetitions per condition). Lastly, and quite uniquely given the experimental setup (BMI coupled with NMES), in the fifth experiment, the subject intended to move, and this resulted in the decoder crossing the threshold that would ultimately evoke a consequence, the tone, but no movement. On different trials, we asked the subject to report either when he first intended to move, or the time of sound onset (50 repetitions per condition). Report type (intention, action, effect) was varied in “mini-runs” of 5 trials, and a full experiment was not run in 1 session, but instead experiments were intermixed between sessions.

### Analyses

Behavioral estimates of each judgment type (intention, action, effect) and different combinations of elements of the intentional chain (Fig 1B, different rows) were coalesced and their circular median was computed. Targeted non-parametric statistics were conducted, as reported in the main text.

Regarding single-unit spiking activity, data files were concatenated across an entire session, filtered with a 300 Hz–30 kHz bandpass filter, and spike sorted via WaveClust [50] in MATLAB. Clusters were then manually inspected for waveform shape and violation of the inter-spike interval. Spikes were binned in 1ms intervals and then spike counts were performed. Correlations with the subjective timing of intention, actions, and effects were computed by Pearson correlation. For firing rate plots in **Fig 3A**, binned spikes were convolved with a Gaussian distribution with a standard deviation of 50 ms. Only units from the first session were kept for the main analyses in main text, to avoid observing units in duplicate.

Multi-unit spiking activity from all 96 recording channels was separated as a function of judgment type and the different combinations of the intentional chain. Spikes were then convolved with a Gaussian distribution (50 ms standard deviation). We summarized this activity by averaging each channel across trials. The onset of evoked MUA was defined as the signal being five times the noise level (standard deviation).

To account for spiking activity of the population of neurons as a whole, we performed a principal component analysis (PCA) on the average firing rate for each condition. We kept the two components that explained the most variance. For statistical analyses, we re-sampled trials (with replacement) 1,000 times, before conducting PCA, and determined the 95% confidence intervals. Power within different frequency bands of the local field potential was determined by wavelet decomposition, and further quantitative analyses of evoked power relative to baseline were computed by SVM (10-fold cross-validated).

Lastly, regarding the neural decoder analyses, the outputs of the decoder were amalgamated as a function of judgment type and different combinations of the intentional chain. Statistical contrasts as specified in the main text were conducted via time-resolved unpaired *t*-tests corrected for multiple comparisons (Bonferroni).

## Supporting information

S1 Fig Evoking hand closure (HC) via M1 decoder. The BMI user was asked to perform HC movements in a self-paced manner, but only during specific intervals (each internal being ~11 s long). These intervals are marked by the red curve being equal to 1. The decoder output spanned between −1 and 1, and would cause movement via NMES upon zero-crossing. Trials were performed in short blocks of five trials, and the figure shows five example blocks of five trials. Decoder output is represented in black. As indicated in the main text, during the course of the entire experiment (12 sessions of ~3–4 hours/session), the user was able to produce HC on 89.2% of requests. On no occasion (0%) was a second action, hand-opening, produced when requesting a HC. However, on occasions, there would be an HC (caused by decoder zero-crossing) without such an explicit request. To estimate the frequency of this occurrence in analyses, we randomly shifted the periods in which a given HC was requested, and estimated how frequently HC occurred during these surrogates. They occurred on 8.1% of surrogate “trials.” Of note, we cannot ascertain whether the participant self-initiated the intention for HC during these periods, even though it was not explicitly requested. The data underlying this figure can be found at https://osf.io/k8r93/.
(TIFF)

S2 Fig Evoked multi-unit activity by environmental effects (tone). **(A)** MUA evoked by the tone, aligned to movement onset. **(B)** MUA evoked by the tone, aligned to the subjective timing of the tone occurring. Shaded areas surrounding the average MUA are S.E.M. No evoked response is observed. The data underlying this figure can be found at https://osf.io/k8r93/.
(TIFF)

S3 Fig Evoked multi-unit activity (MUA) during the full intentional chain (IAE, purple), as well as during cases missing intention (AE, red) or action (IE, green). MUA is aligned to the onset of action, or in the case of no-action trials, to when the decoder reached threshold that would have evoked an action. Shaded areas surrounding the average MUA are S.E.M. Of note, when actions are intended (purple and green), there is a gradual increase in firing rate prior to movement onset. The data underlying this figure can be found at https://osf.io/k8r93/.
(TIFF)

S4 Fig Degrading evoked responses to the subjective onset of intention and action by perturbing timing reports. To establish a degree of certainty/uncertainty surrounding the MUA evoked responses to the subjective timing of intention (red) and action (green), we randomly add or subtract from the reported time a random number of milliseconds (uniform distribution). First within a window of −10 to 10 ms, then from −20 to 20 ms, etc., until −1,000 to 1,000 ms (color gradient). In this manner, we ask: by how much can we perturb/move reported timings without degrading the evoked response to the subjective timing of intention/action. For statistical contrasts, we conclude a response has been degraded once at least 10 time points are different from the original at *p* < 0.01. Acknowledgedly, this is an arbitrary threshold. This statistical threshold is met once timings are perturbed within a ~120 ms window (i.e., −120 ms to +120 ms) surrounding the reported timings of intention and action. The data underlying this figure can be found at https://osf.io/k8r93/.
(TIFF)

S5 Fig Population-level dynamics in primary motor cortex and presence of intentions, actions, and effects. **(A)** Top: Population dynamics on trials with the full intentional chain (black) and trials solely missing intention (red). The two principal components accounting for most of the spiking variance (multi-unit activity) are plotted. Overall these components accounted for 81.9% of multi-unit activity and their shape in latent space showed the circular pattern stereotypical of M1. Hue contrast increases with time. Bottom: Euclidian distance as a function of time from movement onset. The distance in latent space between neural trajectories with or without intention, as a function of time from movement onset. (**B)** and **(C)** follow **(A)**, but for trials with and without action (**B**, green) or environmental effect (**C**, blue). The data underlying this figure can be found at https://osf.io/k8r93/.
(TIFF)

S6 Fig Population-level dynamics in primary motor cortex and the relative subjective timing of intentions, actions, and effects. **(A)** Top: Latent trajectories of trials with intention, perceived relatively early (black) or late (red). Bottom: Euclidian distance between these trajectories, as a function of time from movement onset. **(B)** and **(C)**, are as **(A)**, but separating trials as a function of the subjective timing of actions (**B**, green) and effect (**C**, blue). The data underlying this figure can be found at https://osf.io/k8r93/.
(TIFF)

S7 Fig Detection of intention, actions, and effects in local field power spectrums. **(A)** Power spectra from 0.5 to 30 Hz (y-axis) as a function of time from action onset (x-axis, dashed white line) and whether the trial involved the full intentional chain (top), no intention (second row), no action (third row), or no effect (bottom row). **(B)** Difference in SVM decodability (induced power versus baseline) as a function of whether intention (red), action (green), or effect (blue) was missing from the full intentional chain. The absence of intention is most evident in lower frequency bands and occurs prior to movement onset, and peaks close to movement onset (particularly in the alpha band). The absence of action is most evident in alpha and beta bands, is more transient in nature, and occurs post-movement. The data underlying this figure can be found at https://osf.io/k8r93/.
(TIFF)

S8 Fig Correlation between spikes counts in the second prior to movement and reported time of intention relative to movement. The first eight panels show correlations between spike counts in the second prior to movement (x-axis), and the reported time of intention (y-axis, I-time). The last panel, lower right, shows the distribution of the absolute *r* values. The data underlying this figure can be found at https://osf.io/k8r93/.
(TIFF)

S9 Fig Number of neurons correlating with estimates of intention (I), action (A), and effect (E). Spike counts were performed within a window of 1,000 ms, and correlated with the subjective estimate of the timing of intentions (red), actions (green), and the corollary effect of the action (tone, blue). The spike-count time window was moved 1ms at a time, and the number of neurons demonstrating a significant correlation (*p* < 0.05) is plotted as a function of time (centered in the middle of the time-window; e.g., x = −1,000 ms, means the time-window was from −1,500 ms to −500 ms with respect to movement onset). Results demonstrate a clear over-representation of neurons correlating with the subjective timing of intentions rather than actions or effects. This effect is sustained from ~−2,000 ms to 0 ms post-movement onset. After the movement, no estimate (intention, action, or effect) more frequently correlated with single-unit spiking activity. The data underlying this figure can be found at https://osf.io/k8r93/.
(TIFF)

S10 Fig Individual trial dynamics. **(A)** Four example units across the columns. Top: their average response (black) and logistic fit (red) of this response, as we approach the timing of the reported “urge to move.” Below each average response, example trials from each of the units. These show two broad classes. The first two columns show examples where the averaging of variable timing “step-like” behavior on individual trials results in an average “ramp-like” behavior. The second two example units (columns 3 and 4) show examples where a robust response only exists in a subset of trials (second row), also yielding an average “ramp-like” behavior. **(B)** The average response of single units (top, black, *n* = 34) and individual trials from these units (bottom, red, *n* = 177) were fit to sigmoidal functions (units and trials are kept if *r*^2^ > 0.5). The histograms show the full distributions of estimated t_0_ values (left) indicating when the step occurs (relative to the reported time of the subjective experience of intention), and alpha parameters (right), which is inversely proportional to how “step-like” the sigmoidal functions are. The data underlying this figure can be found at https://osf.io/k8r93/.
(TIFF)

S11 Fig Time to movement onset as a function of decoder area under the curve. Trials were split as a function of their total AUC of the movement decoder and estimate type – timing of intention (red), movement (green), and tone (blue). The AUC did not discriminate for how long it took the decoder to cross the threshold for movement relative to trial onset. The data underlying this figure can be found at https://osf.io/k8r93/.
(TIFF)

S1 Text Supplementary results; supplementary discussion. (DOCX)

## Abbreviations

ACC: anterior cingulate cortex
AUC: area under the curve
BMI: brain–machine interface
HC: hand closing
HO: hand opening
MUA: multi-unit activity
MWP: mean wavelet power
NMES: neuromuscular electrical stimulation
PCA: principal component analysis
SCI: spinal cord injury
SMA: supplementary motor area
SVM: support vector machine
