# Supplementary figures and images for: Neuronal responses in the human primary motor cortex coincide with the subjective onset of movement intention in brain–machine interface-mediated actions
Source: PLoS Biol. 2025 Apr 17;23(4):e3003118. doi: 10.1371/journal.pbio.3003118 (PMC12005534; doi:10.1371/journal.pbio.3003118)

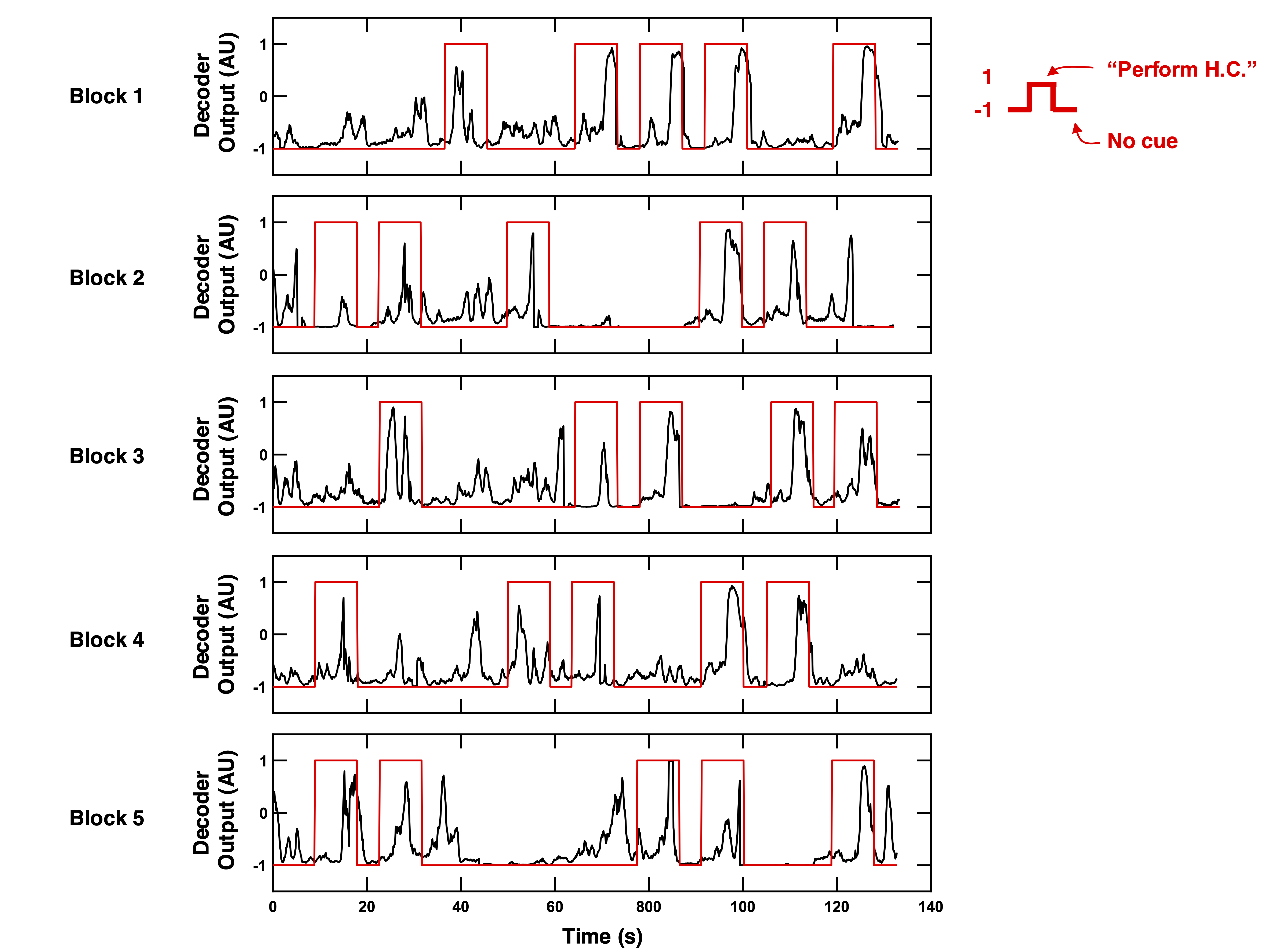

Supplement: S1 Fig — Evoking hand closure (HC) via M1 decoder. The BMI user was asked to perform HC movements in a self-paced manner, but only during specific intervals (each internal being ~11 s long). These intervals are marked by the red curve being equal to 1. The decoder output spanned between −1 and 1, and would cause movement via NMES upon zero-crossing. Trials were performed in short blocks of five trials, and the figure shows five example blocks of five trials. Decoder output is represented in black. As indicated in the main text, during the course of the entire experiment (12 sessions of ~3–4 hours/session), the user was able to produce HC on 89.2% of requests. On no occasion (0%) was a second action, hand-opening, produced when requesting a HC. However, on occasions, there would be an HC (caused by decoder zero-crossing) without such an explicit request. To estimate the frequency of this occurrence in analyses, we randomly shifted the periods in which a given HC was requested, and estimated how frequently HC occurred during these surrogates. They occurred on 8.1% of surrogate “trials.” Of note, we cannot ascertain whether the participant self-initiated the intention for HC during these periods, even though it was not explicitly requested. The data underlying this figure can be found at https://osf.io/k8r93/. (TIFF) [file pbio.3003118.s001.tiff]

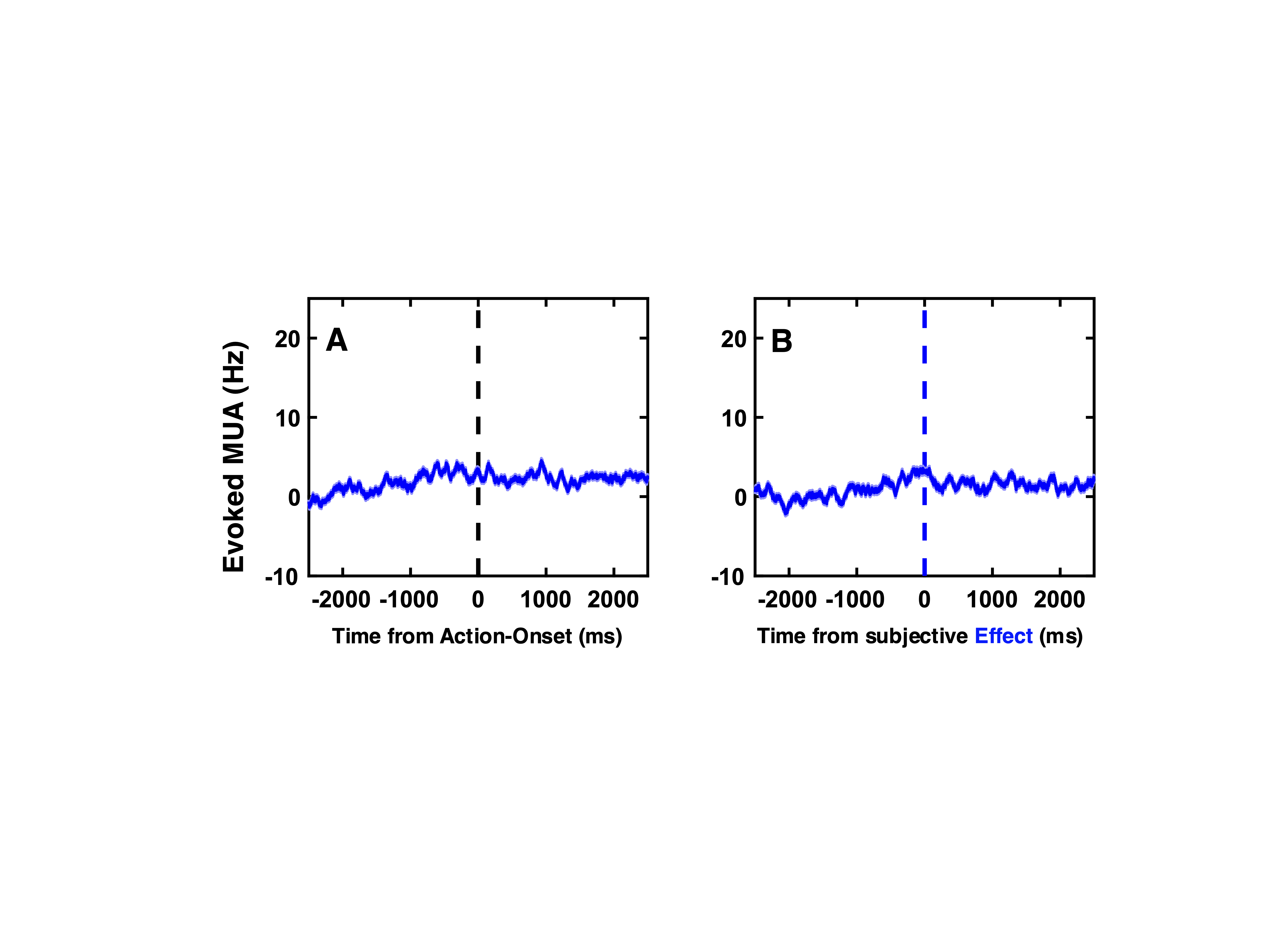

Supplement: S2 Fig — (A) MUA evoked by the tone, aligned to movement onset. (B) MUA evoked by the tone, aligned to the subjective timing of the tone occurring. Shaded areas surrounding the average MUA are S.E.M. No evoked response is observed. The data underlying this figure can be found at https://osf.io/k8r93/. (TIFF) [file pbio.3003118.s002.tiff]

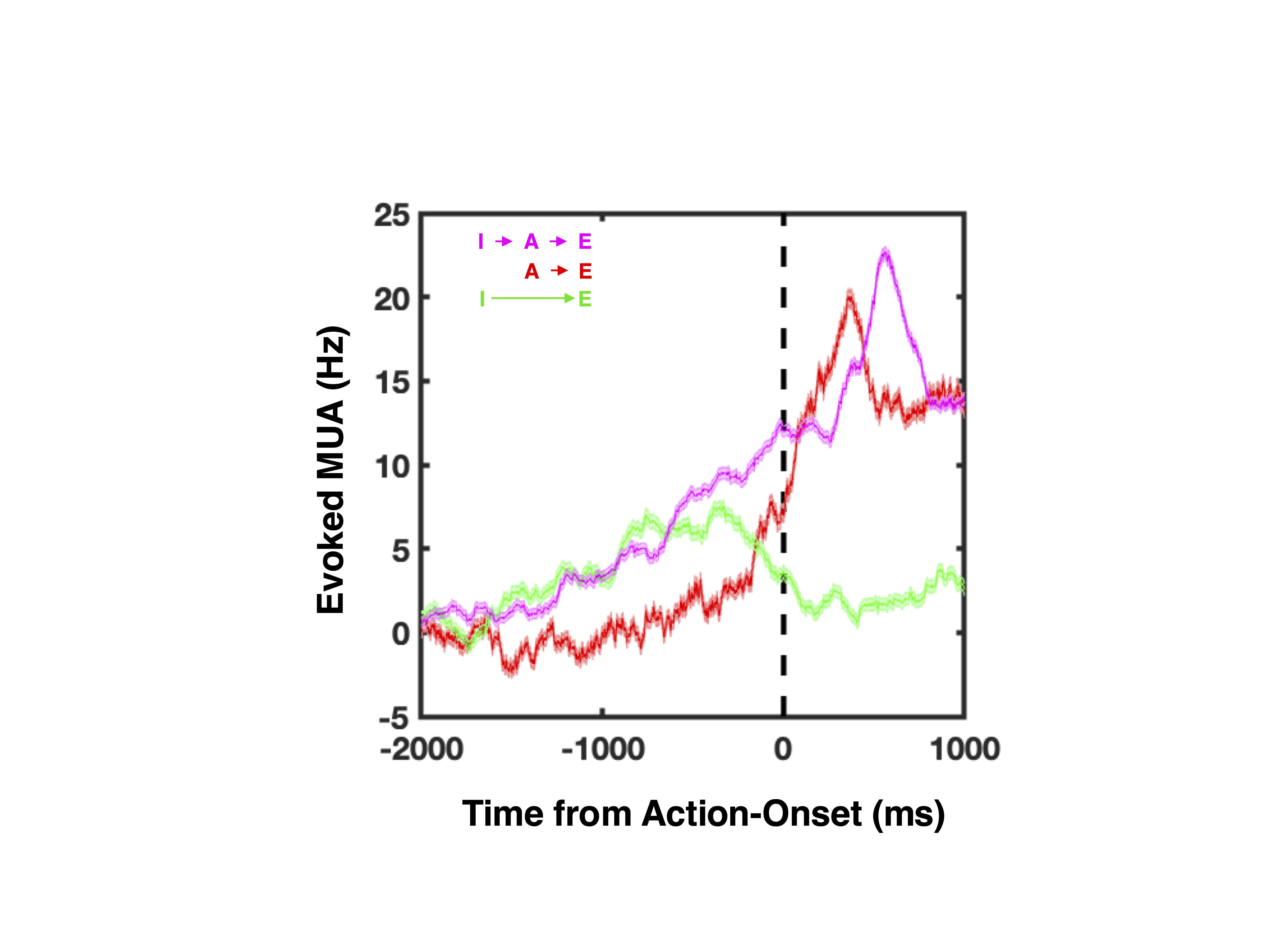

Supplement: S3 Fig — Shaded areas surrounding the average MUA are S.E.M. Of note, when actions are intended (purple and green), there is a gradual increase in firing rate prior to movement onset. The data underlying this figure can be found at https://osf.io/k8r93/. (TIFF) [file pbio.3003118.s003.tiff]

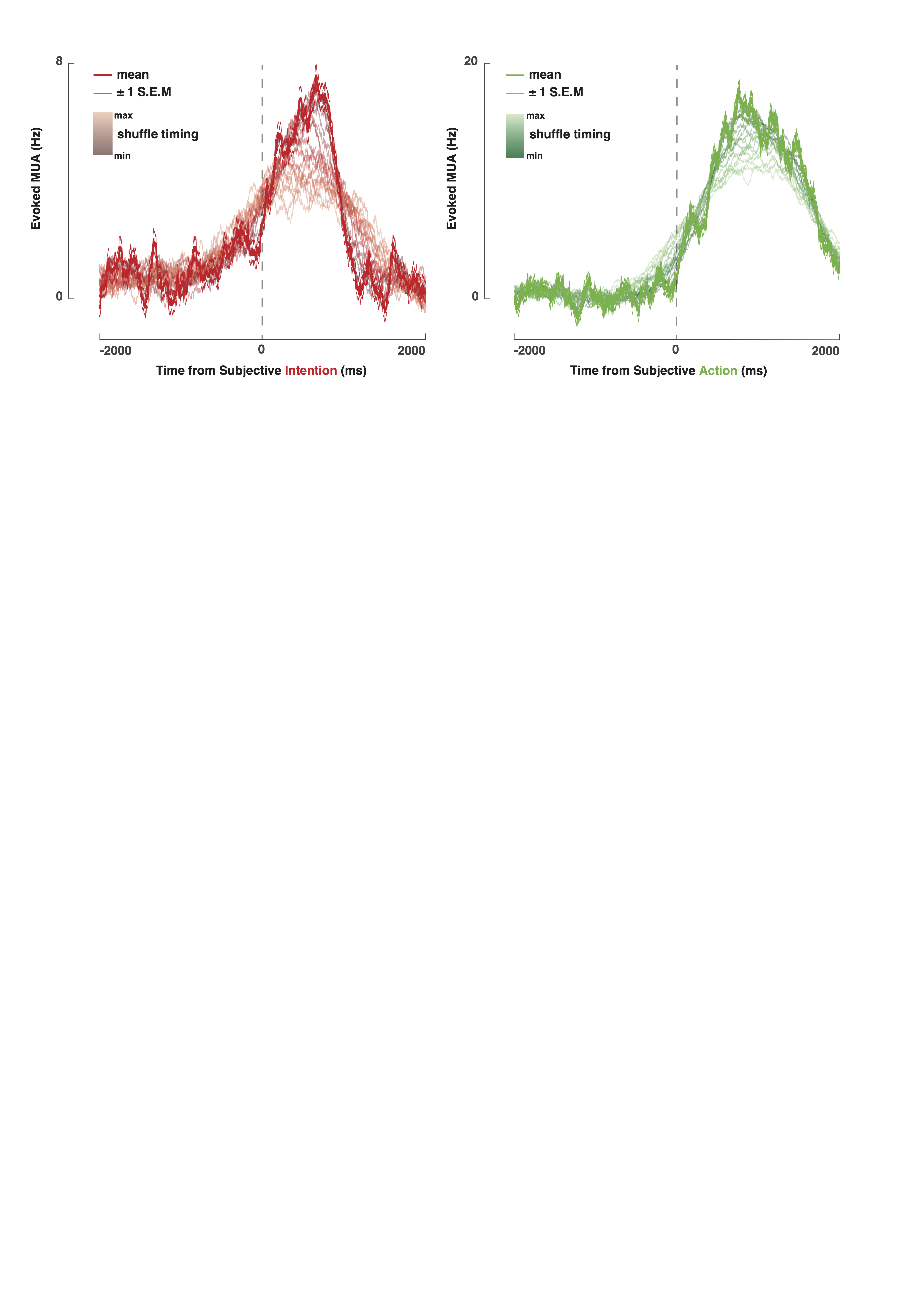

Supplement: S4 Fig — First within a window of −10 to 10 ms, then from −20 to 20 ms, etc., until −1,000 to 1,000 ms (color gradient). In this manner, we ask: by how much can we perturb/move reported timings without degrading the evoked response to the subjective timing of intention/action. For statistical contrasts, we conclude a response has been degraded once at least 10 time points are different from the original at p < 0.01. Acknowledgedly, this is an arbitrary threshold. This statistical threshold is met once timings are perturbed within a ~120 ms window (i.e., −120 ms to +120 ms) surrounding the reported timings of intention and action. The data underlying this figure can be found at https://osf.io/k8r93/. (TIFF) [file pbio.3003118.s004.tiff]

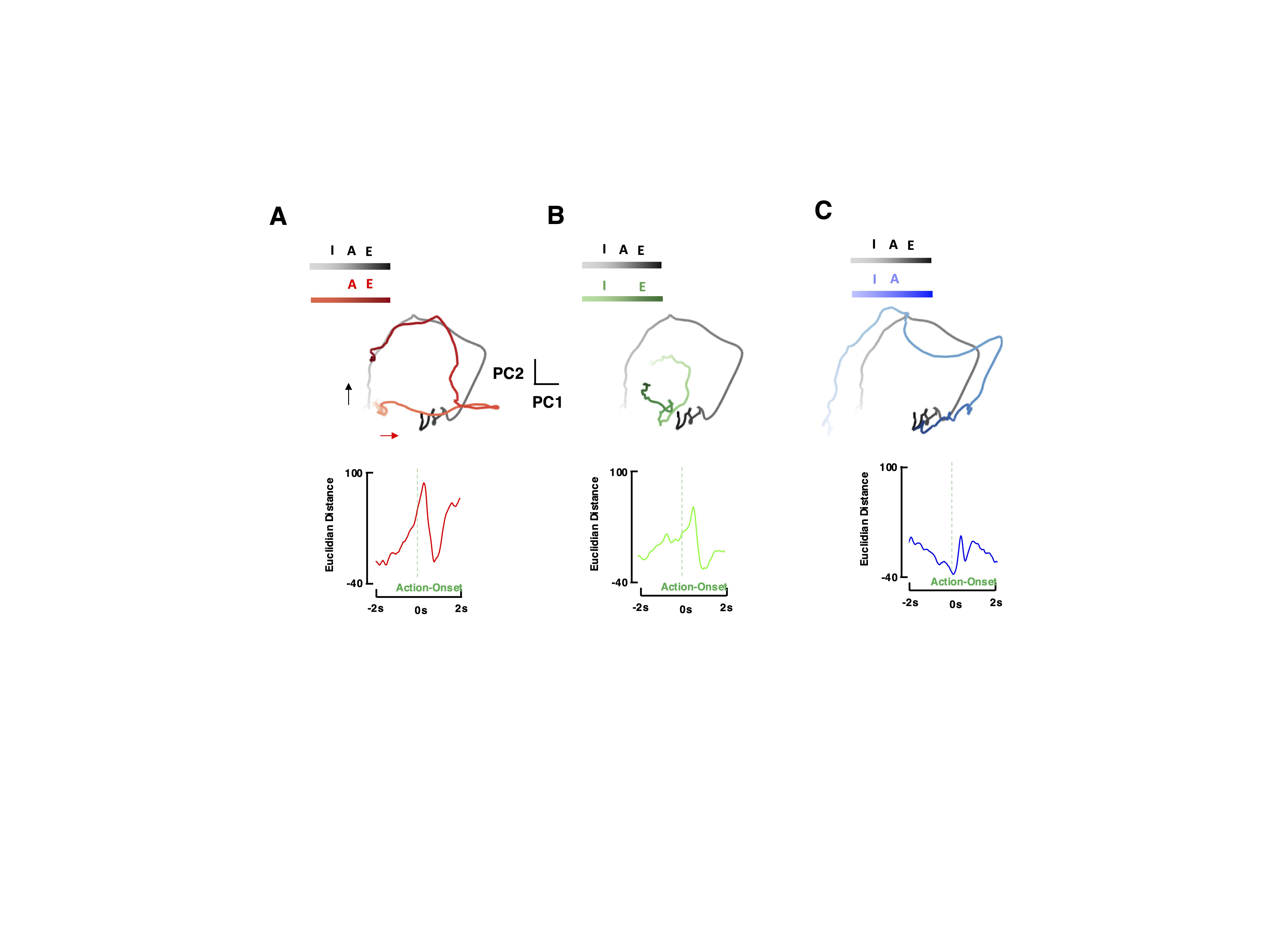

Supplement: S5 Fig — (A) Top: Population dynamics on trials with the full intentional chain (black) and trials solely missing intention (red). The two principal components accounting for most of the spiking variance (multi-unit activity) are plotted. Overall these components accounted for 81.9% of multi-unit activity and their shape in latent space showed the circular pattern stereotypical of M1. Hue contrast increases with time. Bottom: Euclidian distance as a function of time from movement onset. The distance in latent space between neural trajectories with or without intention, as a function of time from movement onset. (B) and (C) follow (A), but for trials with and without action (B, green) or environmental effect (C, blue). The data underlying this figure can be found at https://osf.io/k8r93/. (TIFF) [file pbio.3003118.s005.tiff]

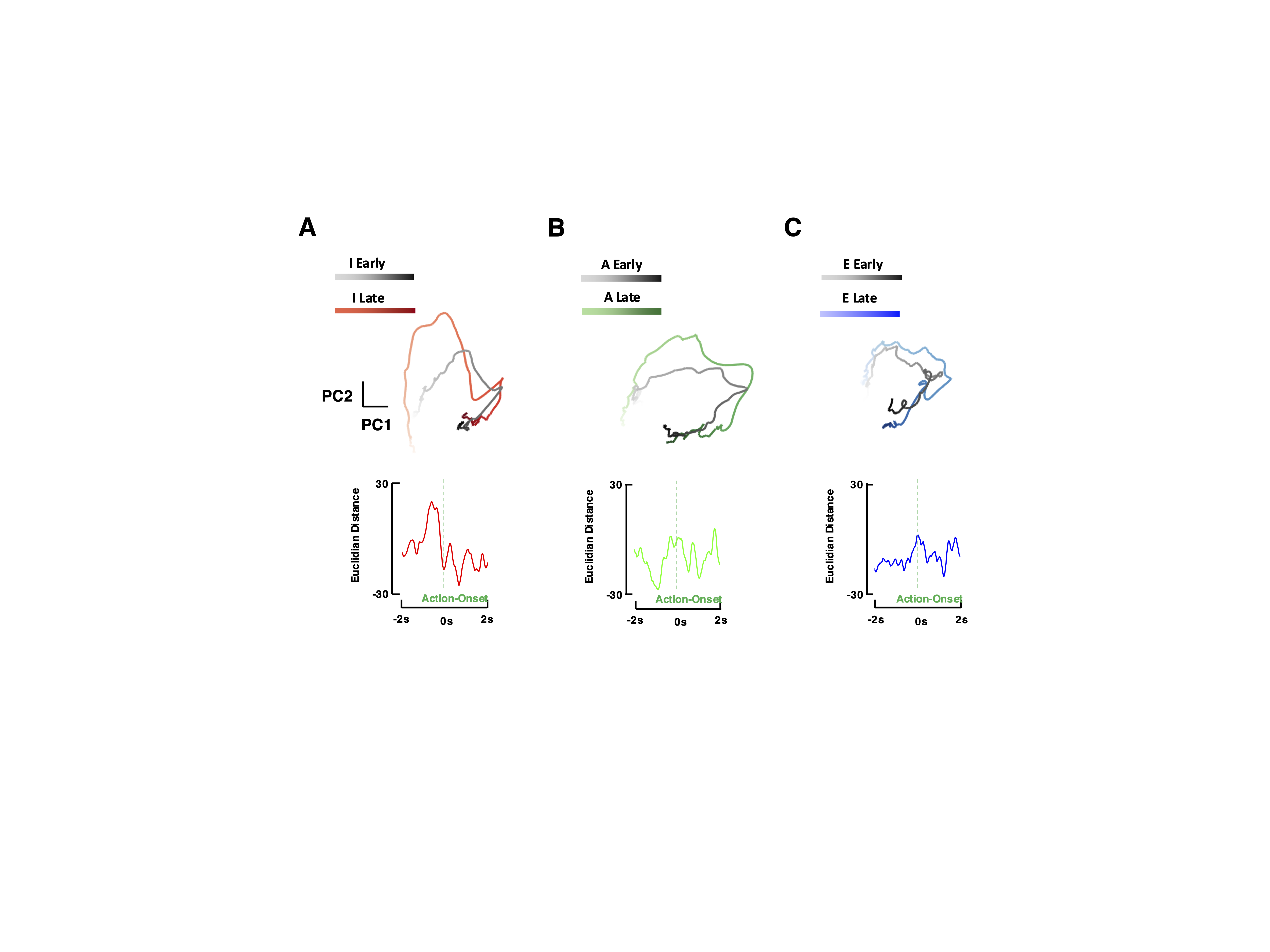

Supplement: S6 Fig — (A) Top: Latent trajectories of trials with intention, perceived relatively early (black) or late (red). Bottom: Euclidian distance between these trajectories, as a function of time from movement onset. (B) and (C), are as (A), but separating trials as a function of the subjective timing of actions (B, green) and effect (C, blue). The data underlying this figure can be found at https://osf.io/k8r93/. (TIFF) [file pbio.3003118.s006.tiff]

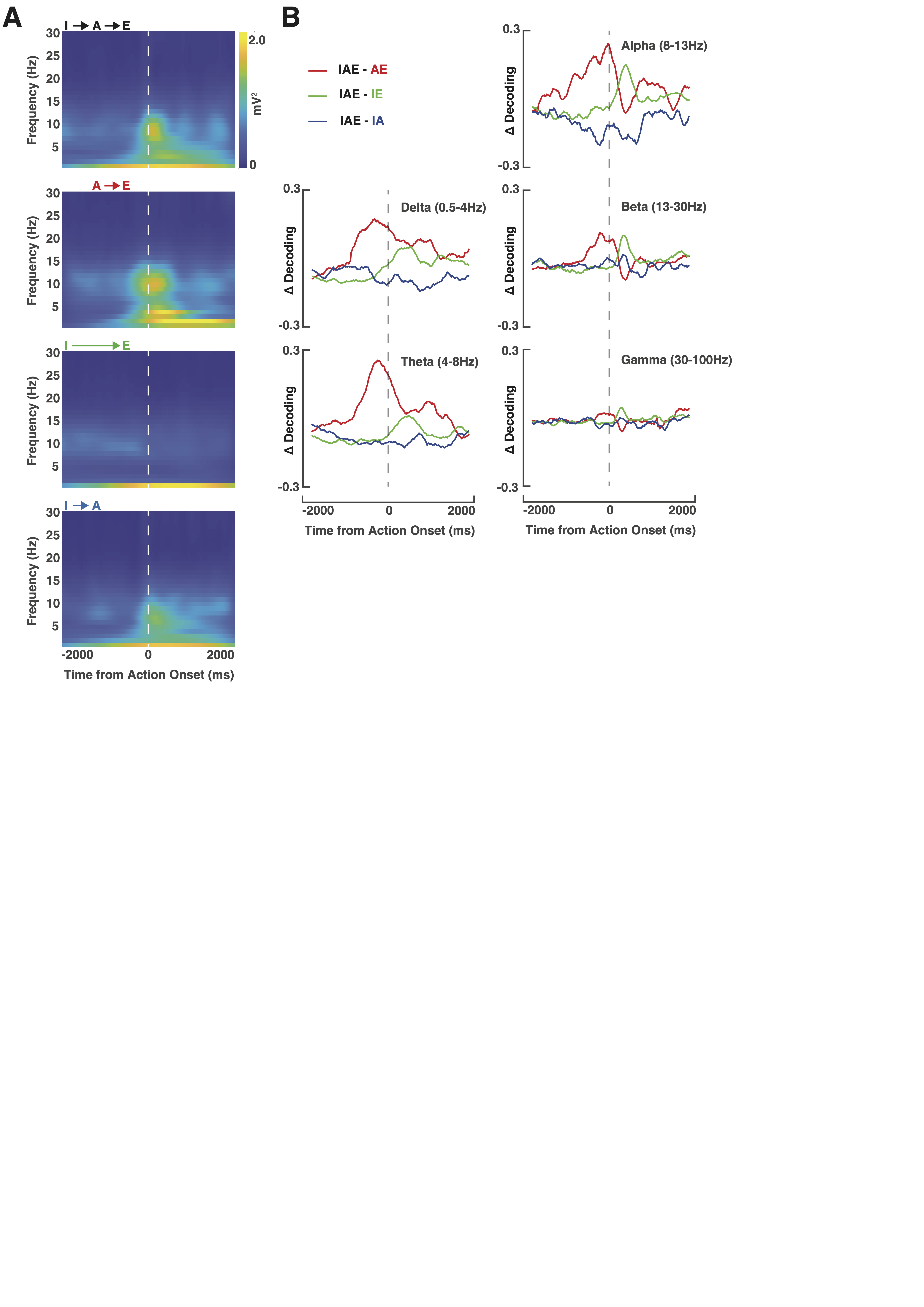

Supplement: S7 Fig — (A) Power spectra from 0.5 to 30 Hz (y-axis) as a function of time from action onset (x-axis, dashed white line) and whether the trial involved the full intentional chain (top), no intention (second row), no action (third row), or no effect (bottom row). (B) Difference in SVM decodability (induced power versus baseline) as a function of whether intention (red), action (green), or effect (blue) was missing from the full intentional chain. The absence of intention is most evident in lower frequency bands and occurs prior to movement onset, and peaks close to movement onset (particularly in the alpha band). The absence of action is most evident in alpha and beta bands, is more transient in nature, and occurs post-movement. The data underlying this figure can be found at https://osf.io/k8r93/. (TIFF) [file pbio.3003118.s007.tiff]

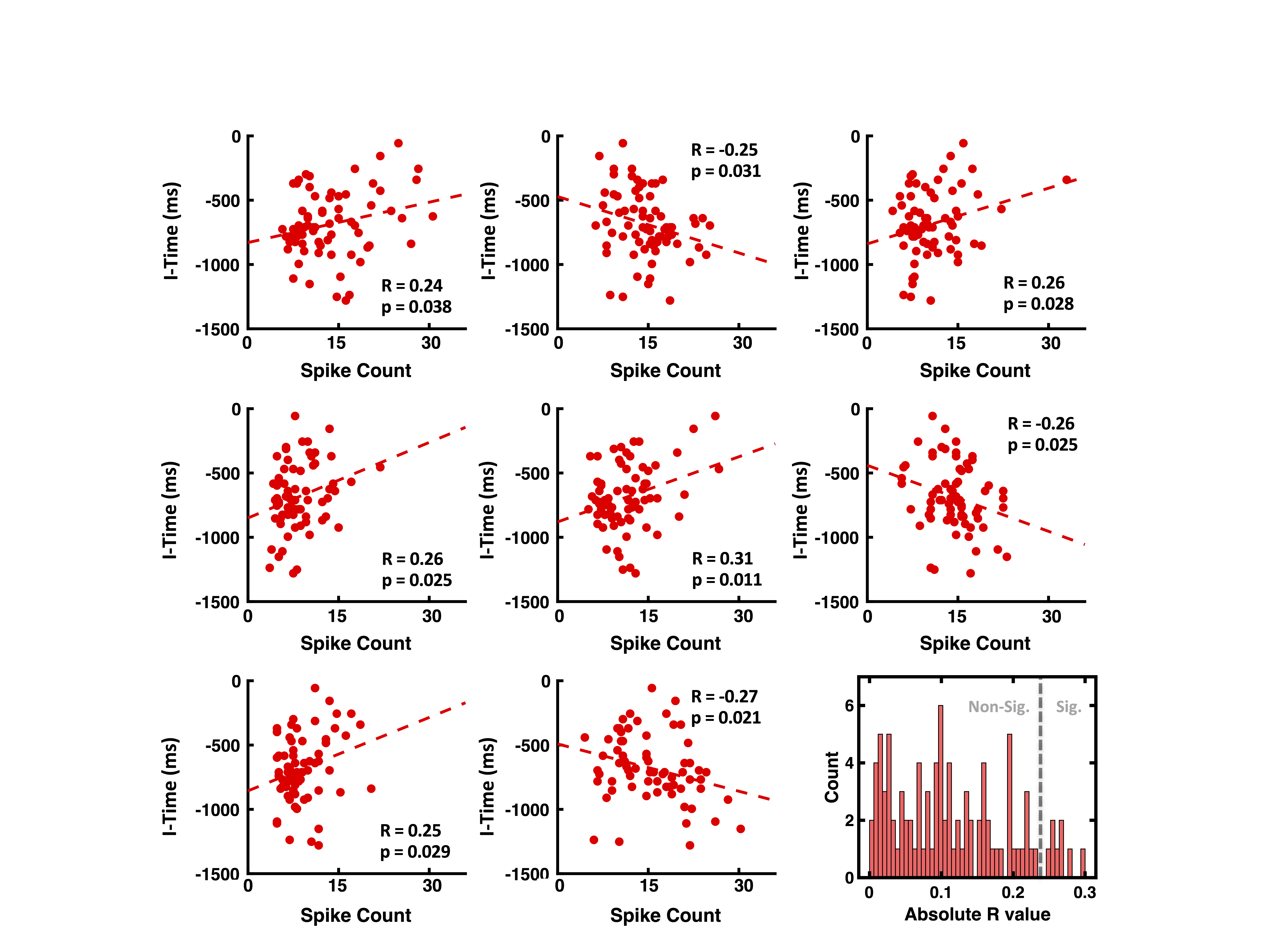

Supplement: S8 Fig — The last panel, lower right, shows the distribution of the absolute r values. The data underlying this figure can be found at https://osf.io/k8r93/. (TIFF) [file pbio.3003118.s008.tiff]

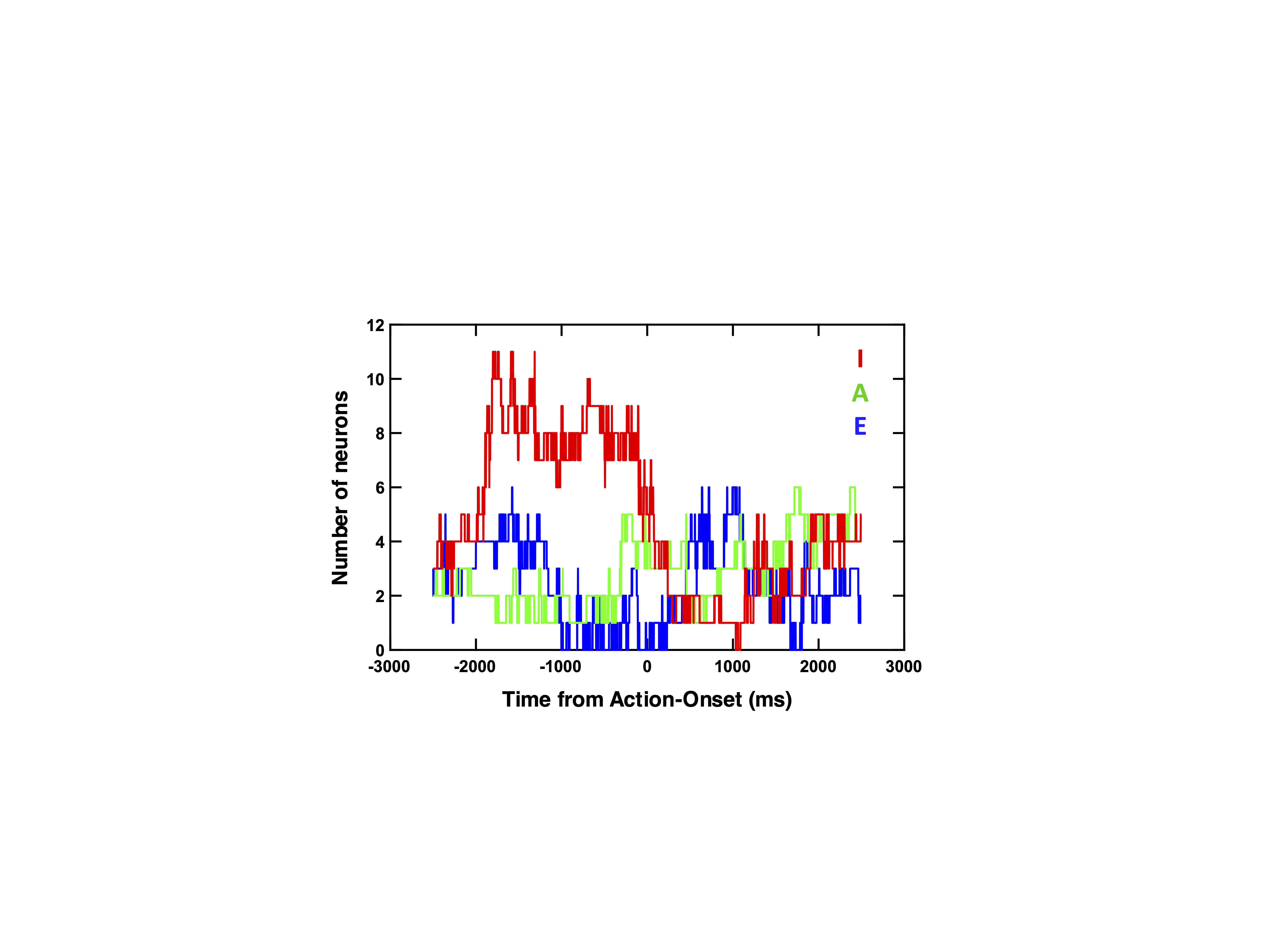

Supplement: S9 Fig — The spike-count time window was moved 1ms at a time, and the number of neurons demonstrating a significant correlation (p < 0.05) is plotted as a function of time (centered in the middle of the time-window; e.g., x = −1,000 ms, means the time-window was from −1,500 ms to −500 ms with respect to movement onset). Results demonstrate a clear over-representation of neurons correlating with the subjective timing of intentions rather than actions or effects. This effect is sustained from ~−2,000 ms to 0 ms post-movement onset. After the movement, no estimate (intention, action, or effect) more frequently correlated with single-unit spiking activity. The data underlying this figure can be found at https://osf.io/k8r93/. (TIFF) [file pbio.3003118.s009.tiff]

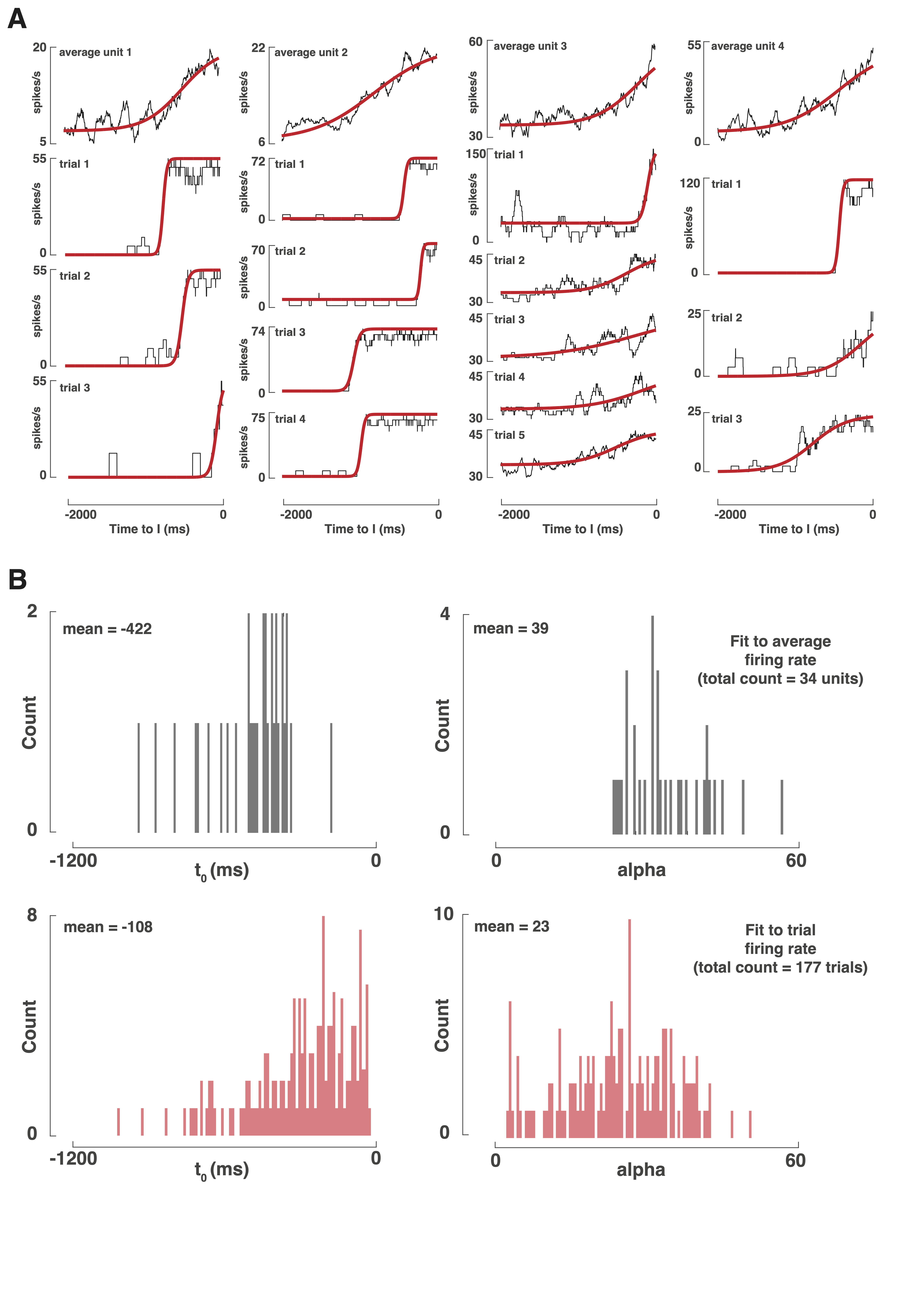

Supplement: S10 Fig — (A) Four example units across the columns. Top: their average response (black) and logistic fit (red) of this response, as we approach the timing of the reported “urge to move.” Below each average response, example trials from each of the units. These show two broad classes. The first two columns show examples where the averaging of variable timing “step-like” behavior on individual trials results in an average “ramp-like” behavior. The second two example units (columns 3 and 4) show examples where a robust response only exists in a subset of trials (second row), also yielding an average “ramp-like” behavior. (B) The average response of single units (top, black, n = 34) and individual trials from these units (bottom, red, n = 177) were fit to sigmoidal functions (units and trials are kept if r2 > 0.5). The histograms show the full distributions of estimated t0 values (left) indicating when the step occurs (relative to the reported time of the subjective experience of intention), and alpha parameters (right), which is inversely proportional to how “step-like” the sigmoidal functions are. The data underlying this figure can be found at https://osf.io/k8r93/. (TIFF) [file pbio.3003118.s010.tiff]

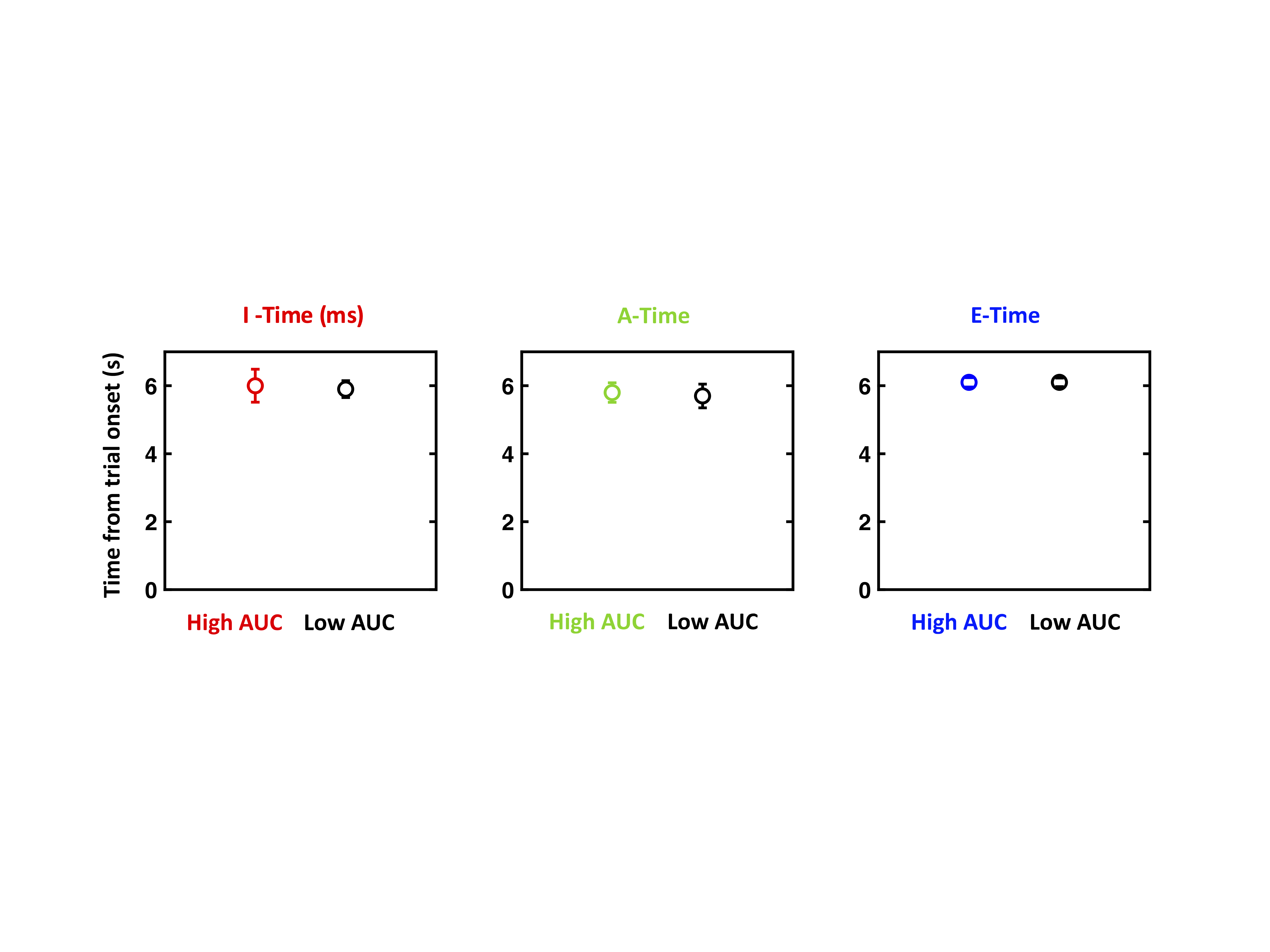

Supplement: S11 Fig — The AUC did not discriminate for how long it took the decoder to cross the threshold for movement relative to trial onset. The data underlying this figure can be found at https://osf.io/k8r93/. (TIFF) [file pbio.3003118.s011.tiff]
