# Supplementary material for: Neuronal responses in the human primary motor cortex coincide with the subjective onset of movement intention in brain–machine interface-mediated actions
Source: PLoS Biol. 2025 Apr 17;23(4):e3003118. doi: 10.1371/journal.pbio.3003118 (PMC12005534; doi:10.1371/journal.pbio.3003118)
Supplement: S1 Text — (DOCX) [file pbio.3003118.s012.docx]

**S1 Text**

Supplementary Results

*Behavior*. The presence of environmental effects did not modulate the perceived timing of intentions or actions. At first glance, this seems contrary to previous reports of intentional binding between actions and effects. Thus, we also asked the BMI participant to report the timing of actions (**Fig 1B, *Row 5***) and effects (**Fig 1B,** *Row 4*) in the absence of other elements of the intentional chain. We compared these estimates with control events in which unintended actions were followed by similar effects (**Fig 1B,** *Row 2*). This showed that actions were perceived as earlier (p = 0.01) and effects were perceived as later (p = 2.2 x 10^-4^) when each event occurred in isolation, as compared to when they occurred in association with each other. This pattern of response reproduces the classic temporal binding effect, but also extends previous reports suggesting that a causal, and not necessarily an intentional relation between actions and effects may be sufficient to engender a degree of attractive pull between one another [1-4]. Nevertheless, the fact that some binding may occur in the absence of intention does not preclude the possibility that an additional, and specific element of binding occurs when intention is additionally present [5, 6].

*Neural Decoding*. As a control, we demonstrate that the AUC of the decoder did not differentiate between the objective timing of action relative to trial onset (as opposed to action onset in the **Main Text**), for neither trials where the BMI participant was asked to estimate the timing of intention, action, or environmental effects (all p > 0.17. **S11 Fig**).

Supplementary Discussion

The timing judgments revealed a strong and previously unmeasured perceived temporal binding between intentions and actions. This resembles the previously described temporal attraction between actions and effects described in the classic intentional paradigm. In fact, in the present BMI-NMES context we also observed, albeit to a lesser extent, binding between actions and effects. This action-effect binding has traditionally been considered to reflect a functional need to bind together sensorimotor events that surround voluntary actions, and that this function may be critical for the normal experience of agency. Temporal binding has now been described in several other experimental contexts, including those without motor intentions. Based on these recent data, several groups [1-4] have suggested that the traditional intentional binding may not be driven by intentional processes per se, but by inferred causality. That is, the repeated occurrence of a specific effect after a specific action may bind the two by multisensory “causal binding” (see [6] for a demonstration of temporal binding as cue integration and **Fig 1B**, *Row 2* vs. *Rows 5 and 6* for corroborative evidence). In turn, the most parsimonious explanation of the intention-action binding herein described may be perceived causality. Moreover, we argue that the stronger temporal binding between intention and action, rather than between action and effect may reflect the lifelong association between intending to move and the resulting movement of the body. That is, there is generally an unequivocal association between intending to move and movement, while the binding between an action and a given effect in the external world depends on the contingent and variable causal structure of the environment. Second, it may be argued that the neuro-cognitive resources demanded by action initiation – particularly in the present BMI-NMES context – outweigh those involved in perceiving the environmental effect and thus may contribute to the greater binding of action toward intention rather than toward the effect. Together, the present behavioral findings demonstrate a new and stronger than the classic form of intentional binding between intention and action.

**References**

1. Buehner, M.J., (2012). Understanding the past, predicting the future: causation, not intentional action, is the root of temporal binding. Psychol Sci, 23, pp. 1490-1497

2. Suzuki K, Lush P, Seth A, Roseboom W. (2019). Intentional binding without intentional action. Psychol Sci,. https://doi.org/ 10.1177/0956797619842191

3. Legaspi, R., and Toyoizumi, T. (2019). A Bayesian psychophysics model of sense of agency. Nat. Commun. 10:4250. doi: 10.1038/s41467-019-12170-0

4. Lush P, Roseboom W, Cleeremans A, Scott R, Seth AK, Dienes Z. (2019). Intentional binding as Bayesian cue combination: testing predictions with trait individual differences. J Exp Psychol Hum Percept Perform.

5. Borhani, K., Beck, B., & Haggard, P. (2017). Choosing, doing, and controlling: Implicit sense of agency over somatosensory events. Psychological Science, 28(7), 882–893. https://doi.org/10.1177/0956797617697693

6. Klaffehn AL, Sellmann FB, Kirsch W, Kunde W, Pfister R. Temporal binding as multisensory integration: Manipulating perceptual certainty of actions and their effects. Atten Percept Psychophys. 2021 Jun 1. doi: 10.3758/s13414-021-02314-0. Epub ahead of print. PMID: 34075560.
